# TAAM refinement on high-resolution experimental and simulated 3D ED/MicroED data for organic mol­ecules

**DOI:** 10.1107/S2053229624005357

**Published:** 2024-06-27

**Authors:** Anil Kumar, Kunal Kumar Jha, Barbara Olech, Tomasz Goral, Maura Malinska, Krzysztof Woźniak, Paulina Maria Dominiak

**Affiliations:** a Biological and Chemical Research Centre Faculty of Chemistry University of Warsaw, ul Żwirki i Wigury 101 02-089 Warszawa Poland; b Centre of New Technologies University of Warsaw, ul S Banacha 2c 02-097 Warszawa Poland; c Faculty of Chemistry University of Warsaw, Pasteura 1 02-093 Warszawa Poland; Rigaku Americas Corporation, USA

**Keywords:** 3D ED, electron diffraction, MicroED, microcrystal, TAAM, IAM

## Abstract

3D electron diffraction (3D ED), or microcrystal electron diffraction (MicroED), has become an alternative technique for determining the high-resolution crystal structures of compounds from sub-micron-sized crystals. Here, we considered l-alanine, α-glycine and urea, which are known to form good-quality crystals, and collected high-resolution 3D ED data on our in-house TEM instrument. In this study, we present a comparison of independent atom model (IAM) and transferable aspherical atom model (TAAM) kinematical refinement against experimental and simulated data. TAAM refinement on both experimental and simulated data clearly improves the model fitting statistics (*R* factors and residual electrostatic potential) compared to IAM refinement. This shows that TAAM better represents the experimental electrostatic potential of organic crystals than IAM. Furthermore, we compared the geometrical parameters and atomic displacement parameters (ADPs) resulting from the experimental refinements with the simulated refinements, with the periodic density functional theory (DFT) calculations and with published X-ray and neutron crystal structures. The TAAM refinements on the 3D ED data did not improve the accuracy of the bond lengths between the non-H atoms. The experimental 3D ED data provided more accurate H-atom positions than the IAM refinements on the X-ray diffraction data. The IAM refinements against 3D ED data had a tendency to lead to slightly longer *X*—H bond lengths than TAAM, but the difference was statistically insignificant. Atomic displacement parameters were too large by tens of percent for l-alanine and α-glycine. Most probably, other unmodelled effects were causing this behaviour, such as radiation damage or dynamical scattering.

## Abstract 2

With the currently avalaible 3D ED data it is possible to observe details of electrostatic potential deformations due to chemical bonding in organic crystals. The deformation signal is strong enough to see the benefits of using a more accurate potential model (TAAM) in achieving a better fit of the model to the experimental data.

## Introduction

Crystal structure provides detailed information about the atomic positions, inter- and intra­molecular inter­actions, and chemical bonding which, in turn, informs about the stability, reactivity, solubility and other physical properties. So far, the structures of small-mol­ecule crystals have been determined using X-ray diffraction methods. In-house single-crystal X-ray diffraction (SCXRD) requires crystals several times larger than the synchrotron radiation source, while suitable crystals for the synchrotron radiation source are typically around 5–10 µm (Nave & Hill, 2005 ▸; Holton & Frankel, 2010 ▸; Gruene *et al.*, 2018 ▸). The crystallization of large and well-ordered single crystals has frequently been a major issue for structure elucidation using the SCXRD method (Terwilliger *et al.*, 2009 ▸; Luft *et al.*, 2011 ▸; Dalle *et al.*, 2014 ▸; Inokuma *et al.*, 2013 ▸). In particular, growing the crystals for the pharmaceutical industry and proteins is a very difficult and time-consuming process (Carpenter *et al.*, 2008 ▸; Gemmi *et al.*, 2019 ▸). The powder X-ray diffraction (PXRD) method has also been used to determine crystal structure when there is a lack of crystals of a suitable size for SCXRD (Thakral *et al.*, 2018 ▸). However, determining crystal structures from PXRD is not straightforward and still remains more challenging compared to SCXRD, especially for larger organic mol­ecules due to many overlapping peaks (Harris & Williams, 2015 ▸).

Recently, electron crystallography has become an attractive alternative technique for determining the crystal structures of small organic mol­ecules, inorganic compounds, metal–organic frameworks (MOFs), peptides and proteins using 3D electron diffraction (3D ED) or microcrystal electron diffraction (MicroED) (Shi *et al.*, 2013 ▸; Beale *et al.*, 2020 ▸; Gruene *et al.*, 2018 ▸; Jones *et al.*, 2018 ▸; Mugnaioli *et al.*, 2020 ▸; Gemmi *et al.*, 2019 ▸). Electrons are charged particles that inter­act strongly with matter. Therefore, it is possible to collect useful 3D ED data on sub-micron-sized crystals, typically of a volume 10^6^ times smaller than that required for SCXRD. Several active pharmaceutical ingredients (APIs) are only available as crystalline powders of sub-micron size that are highly suitable for 3D ED to determine the structures of different crystal forms (Gruene *et al.*, 2018 ▸; Shi *et al.*, 2013 ▸; Nannenga & Gonen, 2019 ▸). 3D ED can be a very fast way to identify several polymorphs of the same compound in a mixture (Jones *et al.*, 2018 ▸; Broadhurst *et al.*, 2020 ▸). The samples can be used directly from synthesis vials without recrystallization and impurities, if present, can also be detected. The comprehensive analysis of 50 organic mol­ecules by Bruhn *et al.* (2021 ▸) revealed the significance of the electron diffraction method. The field is growing rapidly with improvement in terms of methodology development and hardware, such as electron sources, accelerating voltage, detectors and experimental set-ups, including sample preparation, high throughput screening and data collection procedures (Gruene & Mugnaioli, 2021 ▸).

However, electron diffraction measurements are associated with unique challenges. The necessity for high-vacuum con­ditions, typically at pressures of 10^−6^ bar (or lower; 1 bar = 10^5^ Pa), is driven by the susceptibility of the electron beam to absorption by air. Crystals of metal complexes, crystals of hy­drated compounds or biological samples may deteriorate under high vacuum. There are several methods for overcoming this challenge, such as plunge freezing, liquid cell holders, *etc*. (Dobro *et al.*, 2010 ▸; Karakulina *et al.*, 2018 ▸). Another issue in electron diffraction experiments is radiation damage caused by the high energy of the electron beam. While samples in materials science are generally insensitive to radiation damage, biological, pharmaceutical and certain organic compounds exhibit sensitivity to radiation, which often limits their structural analysis (Andrusenko & Gemmi, 2022 ▸). The radiation damage can be reduced by cooling the samples to cryogenic temperatures (Bruhn *et al.*, 2021 ▸).

Electrons inter­act strongly with matter, which causes multiple scattering events, also referred to as dynamical scattering or dynamical effects (Stern & Taub, 1970 ▸). Although the dynamical scattering can be neglected and data can be processed using kinematical approximation to achieve the structure solution, this results in a poor structure model and higher refinement statistics (Broadhurst *et al.*, 2020 ▸; Bruhn *et al.*, 2021 ▸). In the kinematical approximation, it is assumed that the measured intensities are proportional to the square of the structure factor amplitudes. Due to multiple scattering, this linear relationship breaks down. The dynamical effect typically causes stronger reflections to appear weaker, and weaker reflections to appear stronger. The irradiated crystal density and thickness are important factors to consider while modelling the multiple scattering or dynamical effect (Palatinus *et al.*, 2015 ▸; Petříček *et al.*, 2014 ▸; Gruene *et al.*, 2021 ▸). The application of dynamical scattering theory during refienement enables an improved structure model to be achieved, reveals H-atom positions and allows for the assignment of the absolute configuration of the compounds (Klar *et al.*, 2023 ▸; Wang *et al.*, 2022 ▸; Brázda *et al.*, 2019 ▸; Palatinus *et al.*, 2017 ▸).

Higher values of refinement statistics from electron dif­fraction data are also attributed to the use of improper scattering factors. In the standard approach, the Independent Atom Model (IAM) refinement is used, which treats the atoms as spherical and the scattering factors are obtained *via* quantum mechanical calculation considering isolated spherical atoms in their ground state (Brown *et al.*, 2015 ▸; Petříček *et al.*, 2014 ▸). In reality, atoms within crystals have partial charges and their electron density is polarized. Electron scattering is significantly more sensitive to these effects (Yonekura & Maki-Yonekura, 2016 ▸) than X-ray. In X-ray crystallography, it has already been shown that crystal structure refinement may benefit from the use of more accurate scattering approaches, such as Hirshfeld atom refinement (HAR) (Jayatilaka & Dittrich, 2008 ▸; Chodkiewicz *et al.*, 2020 ▸; Kleemiss *et al.*, 2021 ▸) or the transferable aspherical atom model (TAAM) (Brock *et al.*, 1991 ▸; Pichon-Pesme *et al.*, 1995 ▸; Bąk *et al.*, 2011 ▸; Domagała *et al.*, 2012 ▸; Dittrich *et al.*, 2013 ▸; Jha *et al.*, 2020 ▸). TAAM is based on a multipolar representation of electron density. The usage of TAAM in X-ray structure refinement significantly enhances the physical representation of crystals, including the atomic positions and anisotropic atomic displacement parameters (ADPs) (Jelsch *et al.*, 1998 ▸; Jha *et al.*, 2020 ▸; Dittrich *et al.*, 2004 ▸, 2013 ▸; Nassour *et al.*, 2017 ▸). TAAM provides better descriptions of the H-atom positions and precise hydrogen-bond lengths similar to the reference neutron bond lengths, as well as better refinement statistics. Similar improvements in statistics and atomic positions were observed in TAAM refinement on electron diffraction data of carbamazepine (Gruza *et al.*, 2020 ▸) and β-glycine (Jha *et al.*, 2021 ▸). The effects of TAAM refinement, however, were much weaker and less visible in the experimental data than in the simulated data, likely due to the relatively high level of random noise and the presence of strong systematic effects (dynamical effects and radiation damage) in the experimental data. Therefore, we decided to expand our study by collecting novel 3D ED data for model organic mol­ecules, with a focus on obtaining high-quality high-resolution data, while minimizing radiation damage and dynamical scattering.

For this work, we selected l-alanine, α-glycine and urea. These compounds are known to form good-quality crystals with numerous published neutron and high-resolution X-ray diffraction data sets. We collected high-resolution (*d*_min_ = 0.56 Å) 3D ED data on our in-house TEM instrument for these three compounds. We performed the IAM and TAAM kinematical refinement on the experimental data, and the IAM and TAAM refinement on the simulated data, which were based on periodic DFT calculations. Furthermore, we discuss the quality of the measured data by not only the *R*_int_ and *R*1 statistics, but also by the visibility of residual electrostatic potential features characteristic for covalent bonding and lone electron pairs, response of the refinement to change from IAM to TAAM, comparison to trends observed for simulated data and validation of geometry accuracy for non-H and H atoms. Moreover, we also showed how much the TAAM refinement improves the geometry, ADPs, *R* factors and residual potential compared to IAM, when applied to relatively good and complete electron diffraction data.

## Experimental methods

### Materials

l-Alanine, α-glycine and urea were procured from Sigma–Aldrich and were used without further purification. Crystals of l-alanine and urea were grown by slow evaporation using a mixture of solution of ethanol and water (1:1 *v*/*v*) (for l-alanine) or only water (for urea), and the obtained microcrystals were used for the MicroED sample preparation. α-Glycine crystals were used directly, without recrystallization, for the MicroED sample preparation.

### MicroED sample preparation and data collection

**Figure 1:**
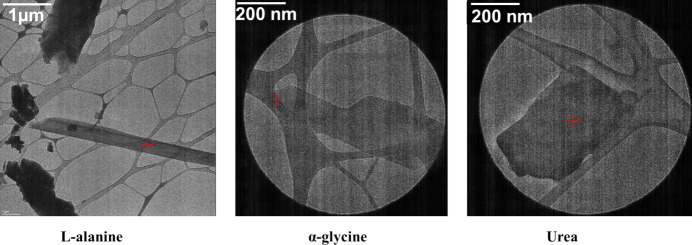
TEM images of microcrystals of l-alanine (left), α-glycine (middle) and urea (right), with the scale bar.

A small amount of each sample was first gently crushed in a mortar and pestle to reduce the crystal size. Grids for MicroED data collection were prepared by directly applying a pinch of the powdered crystals to a freshly glow discharged lacey carbon 200 mesh Cu grid. Following that, the grids were clipped at room temperature (RT) and transferred to the microscope for data collection. Grids were then cooled while the microscope was cooling under vacuum. A Thermo Fisher Scientific Glacios cryo transmission electron microscope (TEM) equipped with a field emission gun operated at 200 kV and a stage holder temperature of 81 K was used for data collection on one single crystal (Fig. 1 ▸) of each compound. The microscope was equipped with a Thermo Fisher Scientific CETA-D detector, an autoloader with 12 grid holders and *EPU-D* software for automated data collection. A 50 µm condenser aperture, spot size 11, and gun lens 8 were set and diffraction data sets were collected under parallel illumination conditions with a very low dose (14.4 e Å^−2^ for l-alanine, 7.2 e Å^−2^ for glycine and 3.6 e Å^−2^ for urea). The crystal was continuously rotated (typically from −60 to +60° for l-alanine and from −50 to +50° for α-glycine and urea) under the parallel beam. The microscope was set in diffraction mode and the camera collected continuously in a rolling shutter mode with hardware binning 2 and exposure time 0.5 s. The collected images were saved in SMV format built in the *EPU-D* software.

### Data processing and refinement details

The unit-cell parameter determination, integration of the reflection intensities and data reduction were performed using *CrysAlis PRO* (Rigaku OD, 2024 ▸). Due to the large discrepancies between the initially determined unit-cell parameters and the literature values (underestimation by >5%), the camera length of the microscope was recalibrated to a new value (657 mm), which led to more accurate unit-cell parameters for all three studied compounds, and the data were reprocessed. The structures were solved in *SHELXT* (Sheldrick, 2015 ▸). All the structural refinements were performed in *OLEX2* (Dolomanov *et al.*, 2009 ▸) using *olex2.refine* in the kinematical diffraction theory approach. The standard spherical model was obtained using the IAM refinement approach. The aspherical TAAM refinement was applied using the MATTS data bank (Jha *et al.*, 2022 ▸; Rybicka *et al.*, 2022 ▸) through the *DiSCaMB* utility discambMATTS2tsc.exe program (Chodkiewicz *et al.*, 2018 ▸) integrated in the *NoSpherA2* module of *OLEX2* (Kleemiss *et al.*, 2021 ▸). In all refinements, the following weighting scheme was applied: *w* = 1/[σ^2^(*F*_o_^2^) + (0.2*P*)^2^], where *P* = (*F*_o_^2^ + 2*F*_c_^2^)/3. All types of refinements were performed without extinction correction to avoid uncontrolled compensation of effects not modelled by the applied model. The atomic coordinates and ADPs (anisotropic for non-H and isotropic for H atoms) were refined freely for all atoms of the three compounds.

## Computational methods

### Reference structures

The crystal structures of l-alanine (Escudero-Adán *et al.*, 2014 ▸; CCDC No. 1009312) and α-glycine (Aree *et al.*, 2012 ▸3; CCDC No. 849663) from X-ray diffraction experimental studies performed at 100 K were used for the theoretical calculations. In the case of urea, the crystal structure from neutron diffraction studies performed at 123 K (Swaminathan *et al.*, 1984 ▸; CCDC No. 1278500) was used for the theoretical calculations.

### Geometry optimization

To obtain the theoretical structure factors, firstly the experimental geometries (atomic coordinates) of l-alanine, α-glycine and urea were optimized with frozen unit-cell parameters by applying periodic DFT calculations using *CRYSTAL17* (Dovesi *et al.*, 2018 ▸). All the calculations were carried out with the B3LYP functional (Civalleri *et al.*, 2008 ▸) and the POB-TZVP basis set (Peintinger *et al.*, 2013 ▸). The B3LYP was augmented with an empirical dispersion term as proposed by Grimme (2006 ▸) and modified for mol­ecular crystals (Civalleri *et al.*, 2008 ▸). A full simultaneous relaxation of the atomic coordinates by means of analytical energy gradients was applied. The level of accuracy in evaluating the Coulomb and exchange series was controlled by five TOLINTEG parameters, for which values of 10^−6^, 10^−6^, 10^−6^, 10^−7^ and 10^−29^ were used. The DFT exchange-correlation con­tribution was evaluated by numerical integration over the unit-cell volume. Radial and angular points of the atomic grid were generated through Gauss–Legendre and Lebedev quad­rature schemes. The condition for the self-consistent field (SCF) convergence was set to 10^−7^ on the total energy dif­ference between two subsequent cycles. The shrinking factors (IS) along the reciprocal lattice vectors were set at 4. The level shifter value was set to 0.6 Hartree. Upon energy convergence, the periodic wave function was obtained.

### Simulated electron diffraction data

The X-ray structure factors were computed through the dedicated module of *CRYSTAL17* from the wave function file (.f9 file) obtained after the geometry optimization step, the set of *hkl* indices (.d3 file) and the anisotropic atomic displacement parameters (ADPs file). The set of *hkl* indices was generated up to a resolution of *d*_min_ = 0.56 Å by the *XD* software (Volkov *et al.*, 2006 ▸) using the *XDHKL* module. For the ADPs file, the ADPs of the non-H atoms were taken from the experimental diffraction data (see *Reference structures*, Section 3.1) for all three compounds. In the case of l-alanine and α-glycine, the anisotropic ADPs for the H atoms were calculated by the SHADE2.1 server (Munshi *et al.*, 2008 ▸) at 100 K, and for urea, the ADPs from neutron diffraction data were used.

Theoretical electron structure factors were computed from theoretical X-ray structure factors by application of the Mott–Bethe formulae using the dedicated *DiSCaMB* utility program (Chodkiewicz *et al.*, 2018 ▸). The theoretical structure factors represented perfect error-free values with constant small values of σ(*F*^2^), which from now on will be called simulated data in this work, and were used to simulate the behaviour of kinematical electron diffraction data during the IAM and TAAM refinements.

### Refinements details

Both the IAM and TAAM refinements on simulated data were performed in the same way as refinements on the experimental data, including the weighting scheme. In the case of the simulated data, the refined parameters were expected to reach the values of the coodinates and ADPs used to compute the data (these values are called target values in this work), as summarized in Table S1 of the supporting information.

## Results and discussion

The electron diffraction data for l-alanine, α-glycine and urea were collected up to a resolution of 0.56 Å, which was satisfactorily high for our studies. Relatively high completeness (71.2–89.5%) and an acceptable mean *I*/σ(*I*) (7.1–10.6) were achieved for all three compounds (see Table 1 ▸). The data were of high quality, probably with little dynamical scattering, as indicated by the relatively low *R*_int_ values (12.97–16.61%) computed for redundant reflections, including symmetry equivalents. The quality of the data for all three compounds seemed to be among the best published so far for organic crystals.

### l-Alanine

#### IAM fitting to the data

**Figure 2:**
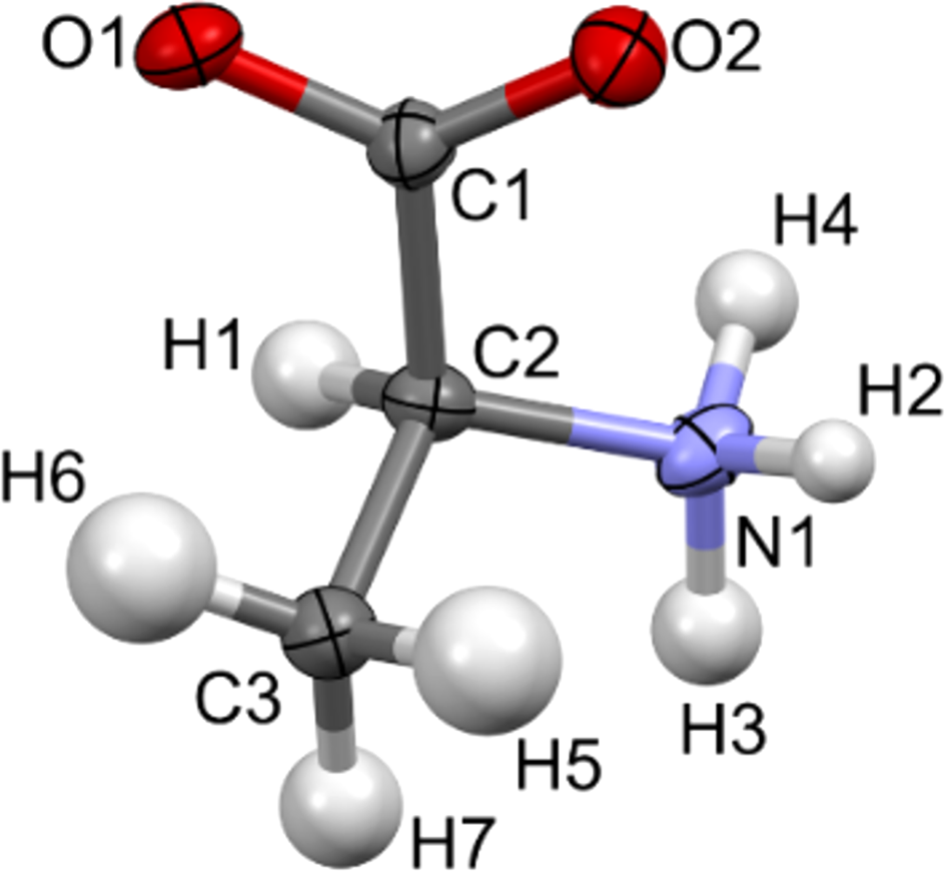
Atomic displacement ellipsoid plot of l-alanine at the 50% probability level after IAM refinement against the experimental data.

**Figure 3:**
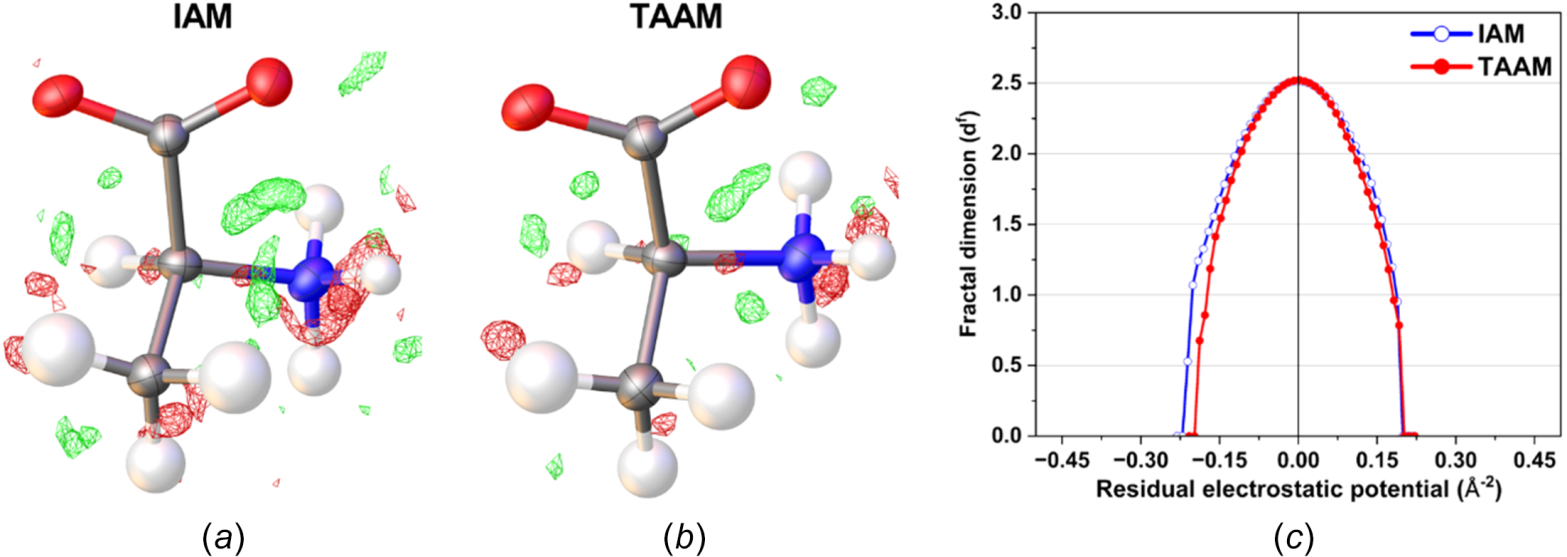
Residual electrostatic potential maps of l-alanine after (*a*) IAM and (*b*) TAAM refinement against the experimental data at ±0.15 Å^−2^ contours (green positive and red negative), and (*c*) fractal dimension plot for the residual potential of the entire unit cell after IAM (blue open circles) and TAAM (red full circles) refinements.

During the IAM kinematical refinement on the experimental data, no restraints or constraints were needed for the coordinates or ADPs. H atoms were visible on residual electrostatic potential maps (Fig. S1 in the supporting information) and, after refinement, it was clear that the amino acid was in its zwitterionic form. The ADPs of the non-H and H atoms were refined anisotropically and isotropically, respectively. After the IAM refinement, all the ADPs of the non-H atoms were positively definite, as shown in Fig. 2 ▸. The *R*1 and *wR*2 values were 13.88 and 37.12%, respectively. The residual electrostatic potential max/min values were 0.207/−0.216 Å^−2^. The shift towards negative values of the residual potential was also reflected in the residual potential map plotted at the ±0.15 Å^−2^ contour [Fig. 3 ▸(*a*)], and confirmed by fractal residual plot [Fig. 3 ▸(*c*), IAM; more details about the meaning of fractal plots are available in the supporting information] to be a global trend for a wide range of values (values larger than *ca* 0.16 Å^−2^ on an absolute scale). The *F*_obs_*versus F*_calc_ plot (Fig. S2, IAM) showed only a slight trend towards overestimation of *F*_calc_ in relation to *F*_obs_, suggesting that there was little dynamical effect present in the data. All these points further confirm that the l-alanine data set seems to be of relatively good quality compared to typical 3D ED/microED data for organic crystals published so far.

Recently published 3D ED data on l-alanine (Khouchen *et al.*, 2023 ▸), collected up to 0.50 Å resolution, showed a completeness of 56%, which is relatively low in comparison to the data presented in this work, and an *R*_int_ value of 11.11%. During the IAM kinematical refinement, all non-H atoms were refined with anisotropic ADPs, but the ADPs and coordinate parameters of the H atoms were constrained. The *R*1 and *wR*2 values were 14.44 and 35.55%, respectively, with residual potential max/min values of 0.23/−0.18 Å^−2^. The published l-alanine data seem to be of not worse quality than ours, apart from the problem with completeness and con­strained H atoms.

#### IAM *versus* TAAM fitting

**Figure 4:**
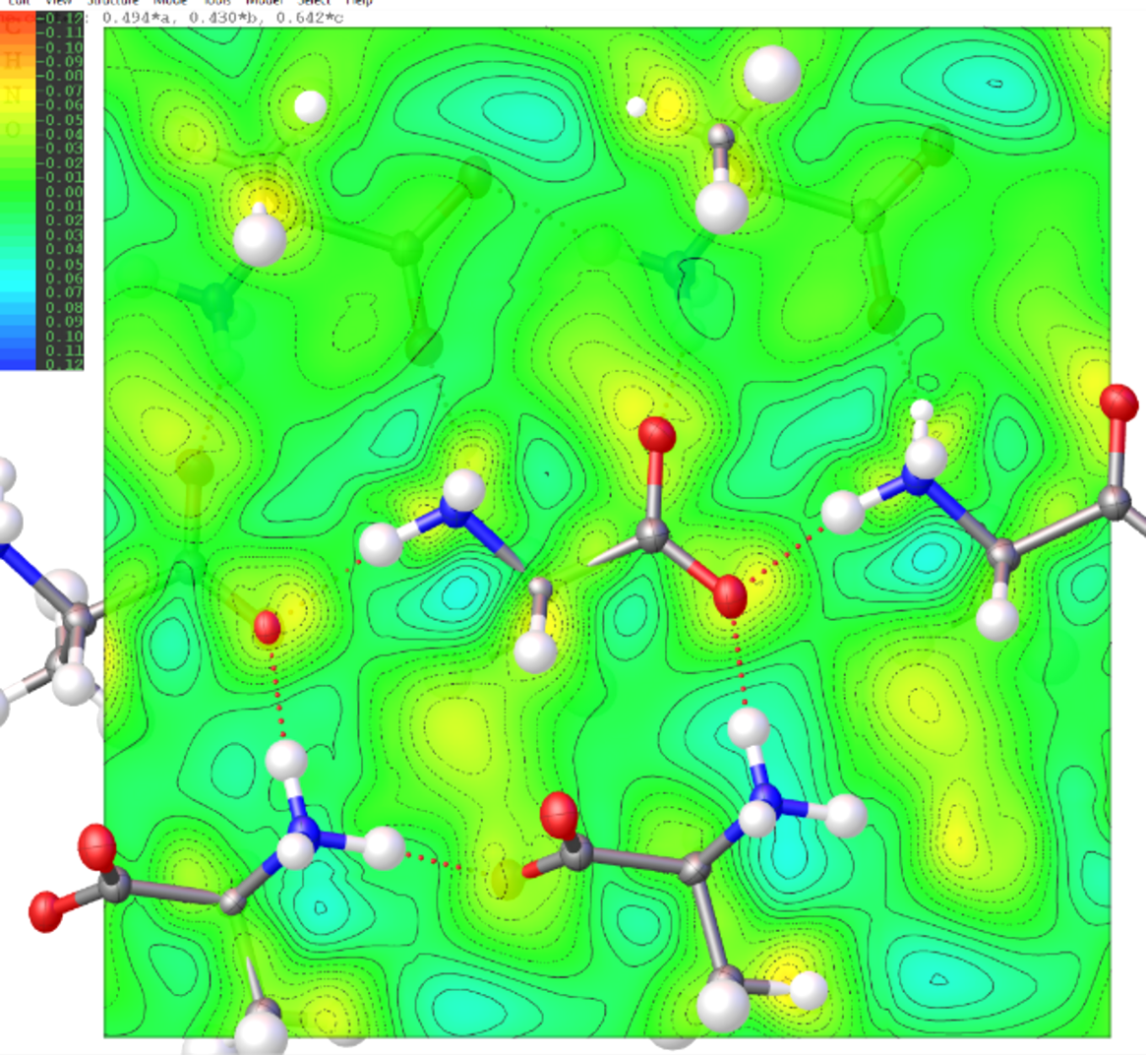
2D Fourier deformation electrostatic potential map (Å^−2^) of the l-alanine crystal computed from TAAM–IAM difference on the structure from the experimental TAAM refinement. The 2D map is plotted on the best plane passing through the N1, C2, C1, O1 and O2 atoms of the central mol­ecule.

The TAAM kinematical refinement was performed against the l-alanine experimental data starting from the crystal structure model obtained from IAM refinement. The TAAM refinement resulted in im­prove­ments of the refinement statistics (Table 1 ▸) and hence the presumably better structure model. The *R*1 and *wR*2 values were improved after TAAM refinement as compared to IAM; the values dropped by 0.75 and 0.54%, respectively, indicating a better fit of the model to the experimental data. The residual potential after TAAM refinement was more featureless compared to IAM, as shown in the residual potential maps [Figs. 3 ▸(*a*) and 3(*b*)]. The visual observation was confirmed by a fractal dimension plot [Fig. 3 ▸(*c*)], where the curve for TAAM is more narrow (less noisy map) and closer to the parabolic shape (more random, less systematically biased map) than for IAM. The effect of the improved fitting was also evident in both the maximum and minimum residual potential values. After TAAM refinement, these values decreased by 0.007 and 0.004 Å^−2^ in absolute value, respectively. Overall, the improvement in the fitting addressed specific negative residual potential regions present in the IAM residual potential, which were subsequently removed by TAAM refinement, as evidenced by the left branch of the fractal dimension plot for TAAM being visibly shifted towards zero compared with IAM. The distribution of residual po­tential seen from the experimental data after the IAM refinement resembled, to some extent, the distributions of deformation electrostatic potential (Fig. 4 ▸), although it was quite noisy. Deformation electrostatic potential maps should illustrate the changes is electrostatic potential appearing due to formation of the chemical bonds and inter­molecular inter­actions, and are computed as a difference between the crystal electrosatic potential and the electrostatic potential computed from superposition of the electrostatic potential of neutral spherical atoms. The deformation potential maps in this work were computed as the TAAM–IAM Fourier maps, to take into account the influence of resolution and atomic displacement parameters on the map. The TAAM–IAM maps show the effects of the formation of chemical bonds on the electrostatic potential, as modelled by TAAM, but not polarization due to inter­molecular inter­actions.

The *F*_obs_*versus F*_calc_ plots were similar for the IAM and TAAM refinements (Fig. S2). *F*_calc_ from TAAM tended to be more like *F*_obs_, especially for the most intense reflections. The *F*_obs_*versus F*_calc_ plot for TAAM strengthens the suggestion that there were minimal dynamical effects present in the data.

The observations regarding the fitting statistics done for the l-alanine experimental data follow the results from the simulated data. The *R*1 value dropped by 1.61% after the TAAM refinement against the simulated data as compared to IAM (Table S2). The residual potential after TAAM refinement was visibly more featureless as compared to IAM [Figs. S3(*a*) and S3(*b*)], and the max/min residual potential values after TAAM became smaller by 0.005/0.079 Å^−2^ and closer to each other on an absolute scale. The fractal dimension plots showed the same trends as for the experimental data [Fig. S3(*c*)], though the dominance of negative values in the IAM residual potential was much more visible and both fractal dimension curves, for IAM and TAAM, were narrower com­pared to the experimental results, due to lack of noise (random or systematic due to dynamical scattering or other effects) in the simulated data.

### α-Glycine

#### IAM fitting to the data

**Figure 5:**
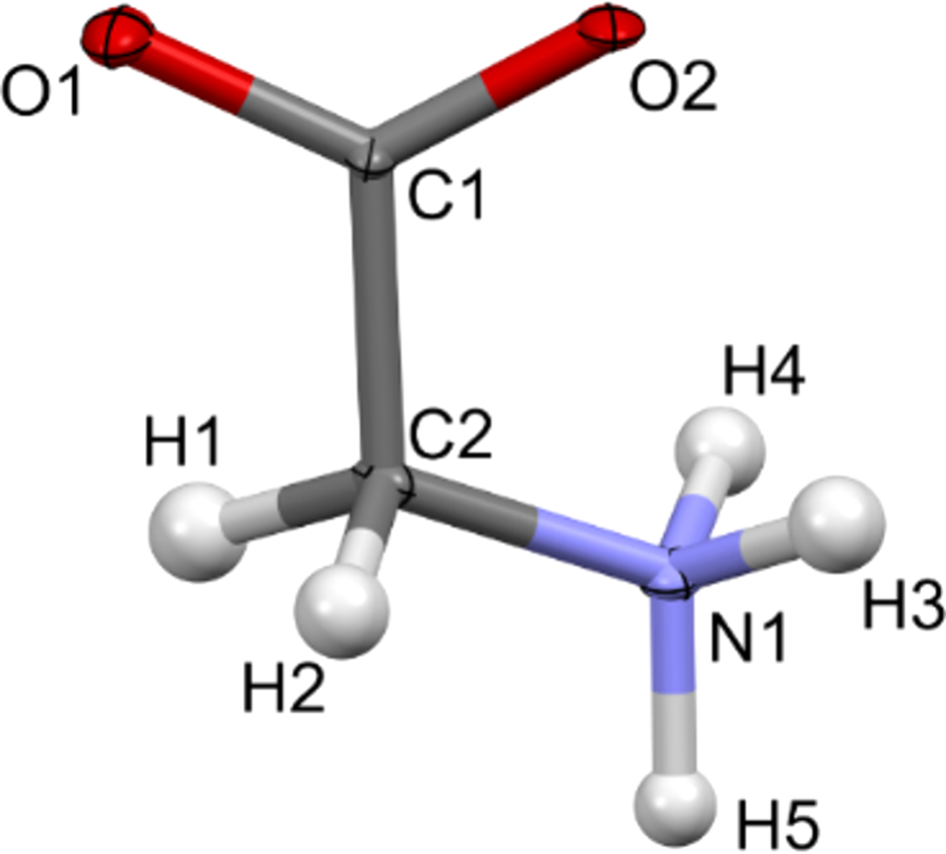
Atomic displacement ellipsoid plot of α-glycine at the 50% probability level after IAM refinement against the experimental data.

**Figure 6:**
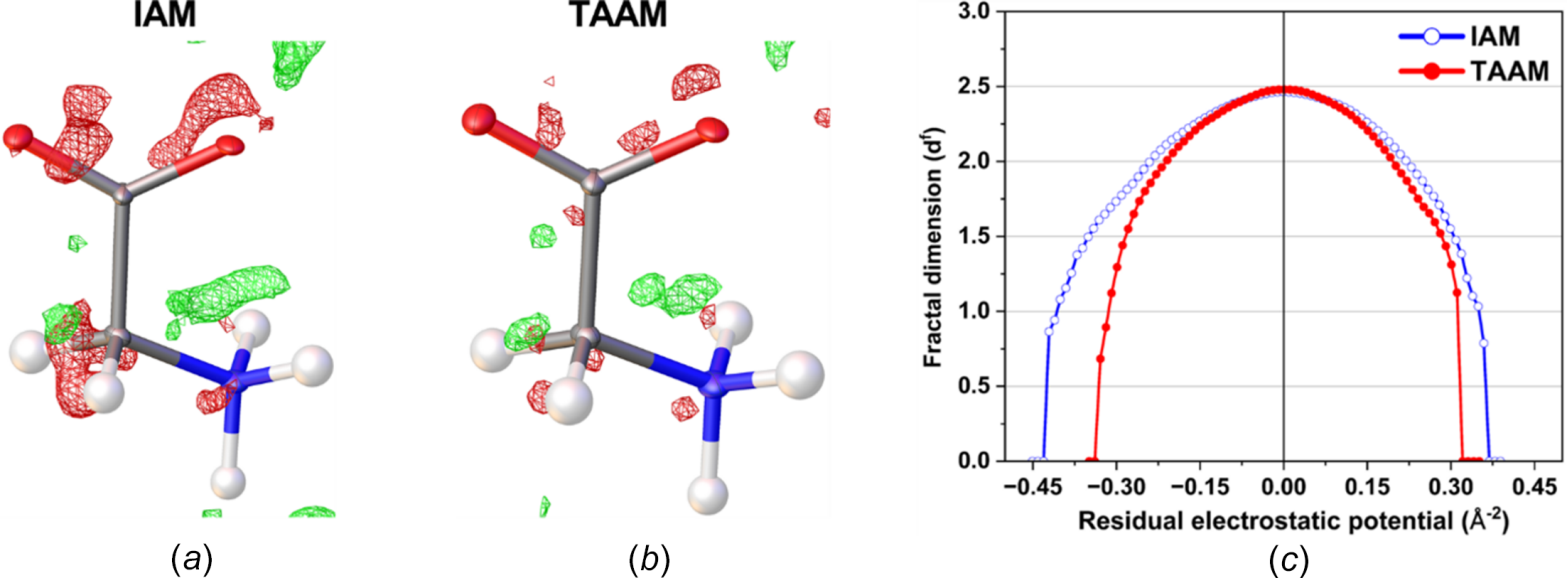
Residual electrostatic potential maps of α-glycine after (*a*) IAM and (*b*) TAAM refinement against the experimental data at ±0.27 Å^−2^ contours (green positive and red negative), and (*c*) fractal dimension plot for the residual potential of the entire unit cell after IAM (blue open circles) and TAAM (red full circles) refinements.

As for l-alanine, no restraints or constraints on the coordinates for all atoms, anisotropic ADPs for non-H atoms or isotropic ADPs for H atoms were necessary during the IAM refinement of the α-glycine structure against the experimental 3D ED data. H atoms were visible on residual electrostatic potential maps (Fig. S4) and, after refinement, it was clear that the amino acid was in its zwitterionic form. After the IAM refinement, the ADPs of the non-H atoms were positively definite, as shown in Fig. 5 ▸. The *R*1 and *wR*2 values were 15.96 and 35.78%, respectively, slightly greater than for l-alanine (Table 1 ▸). The residual po­tential was noisier than for l-alanine [wider fractal curve; Fig. 6 ▸(*c*)] and more visibly dominated by negative values [Figs. 6 ▸(*a*) and 6(*c*)], from the ±0.15 Å^−2^ values and larger on an absolute scale. The max/min residual potential values were 0.366/−0.434 Å^−2^. Most of the negative residuals were located close to the non-H atoms or around the covalent bonds, as shown in Fig. 6 ▸(*a*). The *F*_obs_*versus F*_calc_ plot (Fig. S5, IAM) showed some trend towards overestimation of *F*_calc_ in relation to *F*_obs_, being visibly bigger for strong reflections. For some strong reflections, the *F*_calc_ was *ca* twice as large as *F*_obs_. Compared to l-alanine, the deviation of the plot from the *F*_obs_ = *F*_calc_ line was much bigger, suggesting there were more dynamical effects present in the data.

The first published 3D ED data for α-glycine (Broadhurst *et al.*, 2020 ▸) were collected up to a resolution of 0.70 Å and showed a completeness of 85% after merging data for six crystals, and an *R*_int_ value of 31.8%. The crystal structure model was refined with restraints and constraints. The *R*1 and *wR*2 values were 21.9 and 51.8%, respectively, which are relatively higher than the statistics for the α-glycine data from this work. The residual potential max/min values were 0.23/−0.25 Å^−2^.

In another article (Klar *et al.*, 2023 ▸), the 3D ED data had a completeness of 40.0% at a resolution of 0.59 Å. The IAM kinematical refinement was carrried out with five restraints. The *R*1 and *wR*2 values were 13.6 and 15.9% respectively. The residual potential max/min values were 0.412/−0.420 Å^−2^. The data seem to be of a quality comparable to the data from this work, although with significantly lower completeness. After dynamical refinement, the *R*1 and *wR*2 values were 6.8 and 8.8%, respectively, and the residual potential max/min values were 0.197/−0.162 Å^−2^. This demonstrated the effectiveness of the dynamical approach in achieving a better fit of the model to the data.

#### IAM *versus* TAAM fitting

The TAAM refinement against experimental electron diffraction data for α-glycine improved the fitting of the model to the data. After TAAM refinement, the *R*1 and *wR*2 values became smaller by 1.25 and 1.53%, respectively (Table 1 ▸). The max/min residual potential values became smaller by 0.044/0.105 Å^−2^ on an absolute scale. The residual potential map was much cleaner after TAAM refinement compared to IAM, as shown in Fig. 6 ▸(*b*). The fractal dimension curve for TAAM confirmed that the TAAM refinement led to less noisy residual potential (more narrow curve) and allowed the modelling of some of the unmodelled potential by IAM negative residuals (more symmetric curve), but still the TAAM residual potential showed slightly more negative than positive features [Fig. 6 ▸(*c*)].

**Figure 7:**
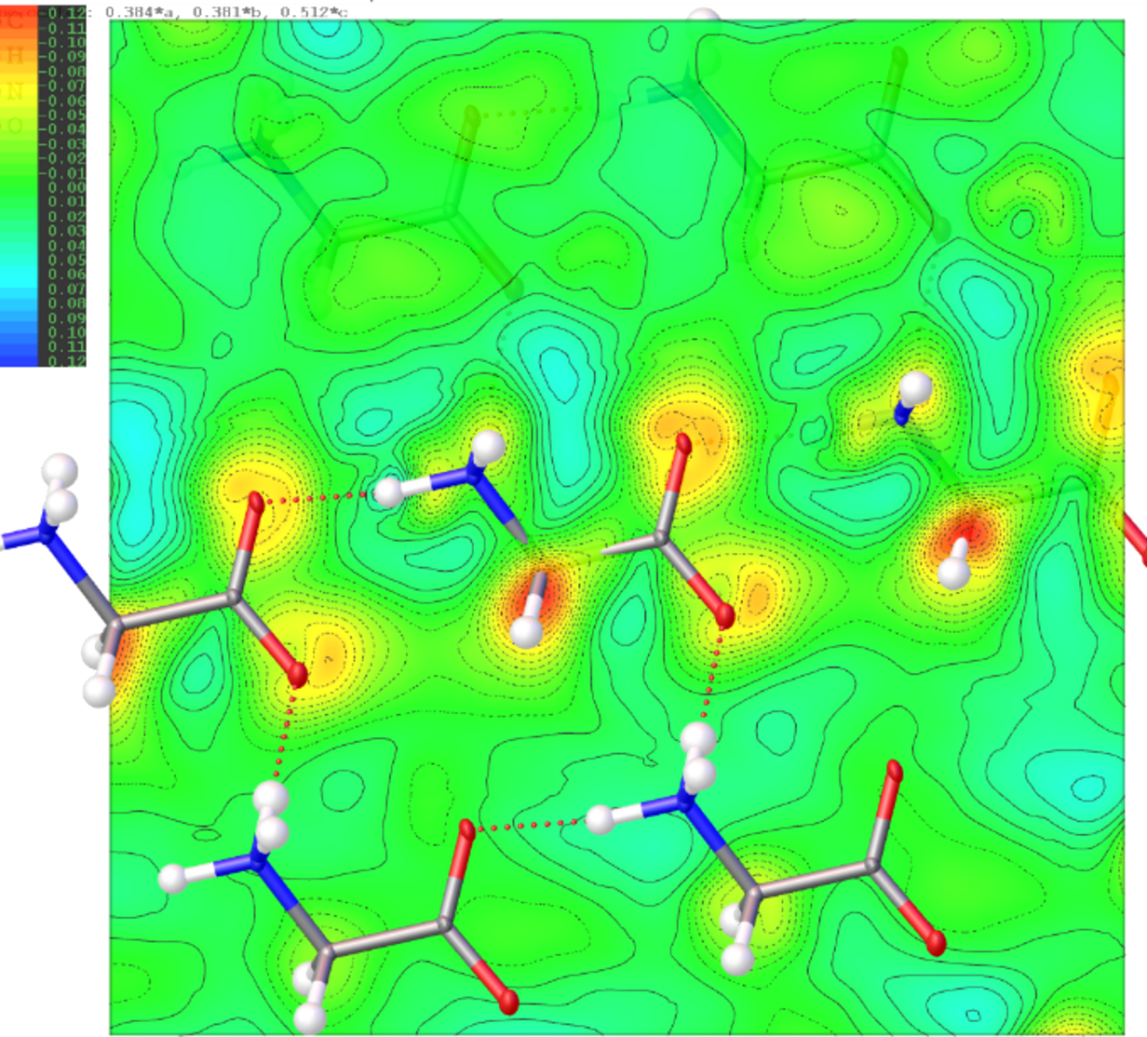
2D Fourier deformation electrostatic potential map (Å^−2^) of the α-glycine crystal computed from TAAM–IAM difference on the structure from experimental TAAM refinement. The 2D map is plotted on the best plane passing through the N1, C2, C1, O1 and O2 atoms of the central mol­ecule.

In the case of α-glycine, the distribution of the experimental residual potential after IAM refinement [Fig. 6 ▸(*a*)] more closely resembled the deformation potential (Fig. 7 ▸) compared to l-alanine. This observation suggests that the data for α-gly­cine contained more chemical information than what IAM can adequately model. The small negative features remaining in the residual potential after TAAM refinement sug­gested that either (i) TAAM alone was insufficient and some effects affecting the electrostatic potential of α-glycine crystal were not accounted for, like polarization due to inter­molecular inter­actions, or (ii) TAAM was appropriate, and other factors beyond modelling the potential played an important role here, such as dynamical scattering.

The *F*_obs_*versus F*_calc_ plots were similar for the IAM and TAAM refinements (Fig. S5). Again, *F*_calc_ from TAAM tended to be closer to *F*_obs_, especially well visible for strong reflections. After TAAM refinement, the tendency to overestimate *F*_calc_ in relation to *F*_obs_, traditionally attributed to the presence of dynamical effects, was smaller but still visible.

The behaviour of fitting characteristics when going from the IAM to TAAM refinement of α-glycine against experimental data was confirmed by the simulated data. For the simulated data, *R*1 dropped by 1.46% after the TAAM refinement as compared to IAM (Table S2). The minimum residual potential peak was lower by 0.066 Å^−2^ in absolute value, but the maximum peak was higher by 0.031 Å^−2^. Nevertheless, the maximum and minimum peaks from the TAAM refinement became more symmetrically distributed around zero than those from IAM. Furthermore, the residual potential map after TAAM refinement was more featureless as compared to IAM [Figs. S6(*a*) and S6(*b*)], though some small non-random features remained after the TAAM refinement. The presence of some systematic effect in the simulated data not accounted for by TAAM was also reflected in the fractal dimension plot, where the curve for TAAM was still not parabolic [Fig. S6(*c*)]. This suggests that, also in the case of the experimental data, we see the insufficiencies of TAAM.

### Urea

#### IAM fitting to the data

**Figure 8:**
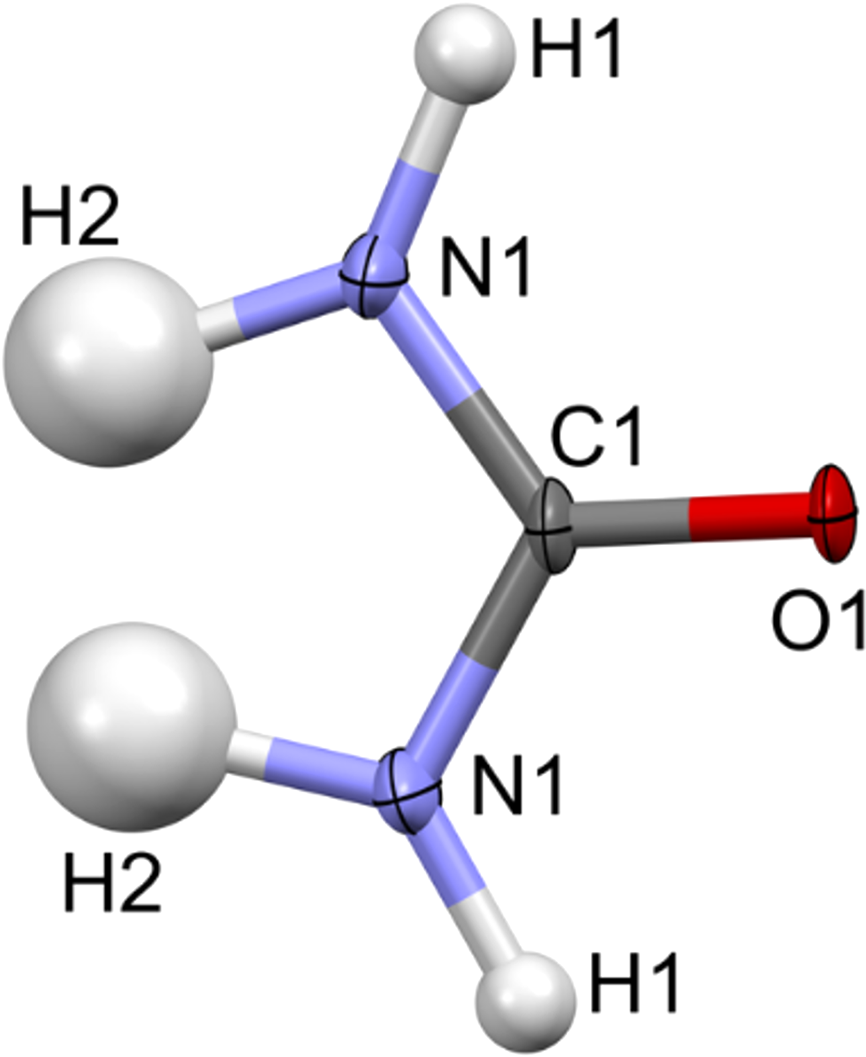
Atomic displacement ellipsoid plot of urea at the 50% probability level after IAM refinement against the experimental data.

**Figure 9:**
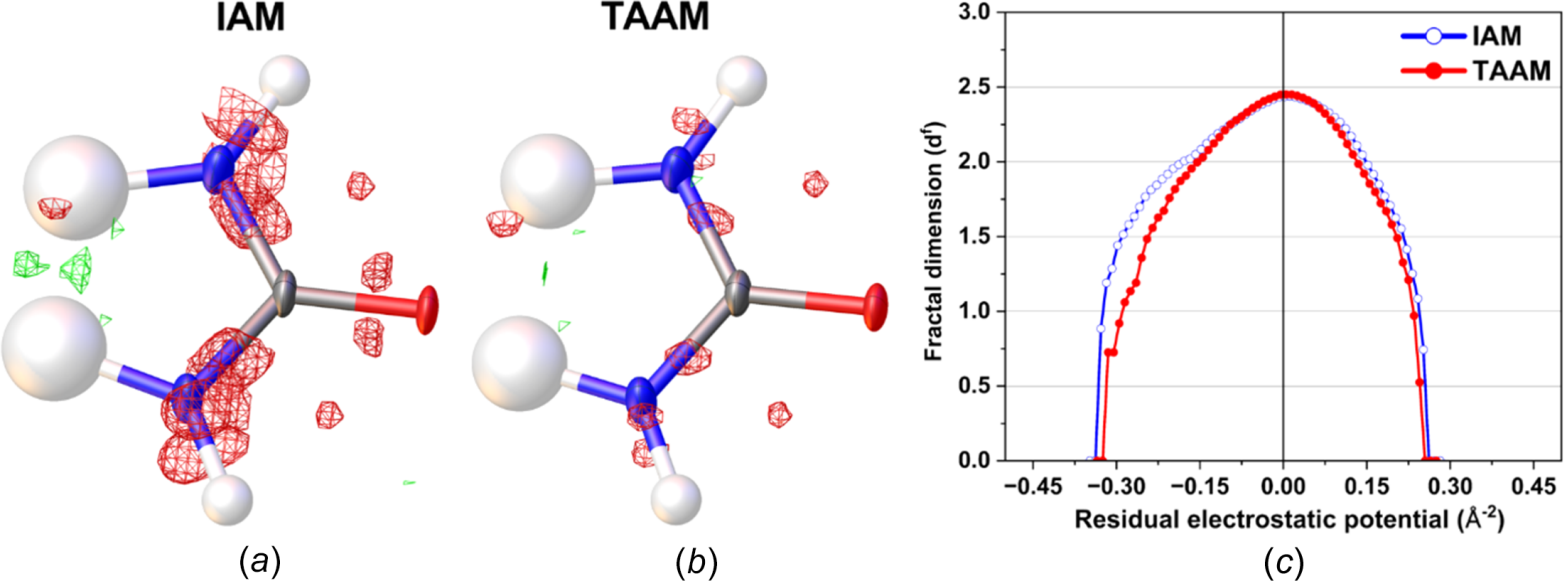
Residual electrostatic potential maps or urea after (*a*) IAM and (*b*) TAAM refinement against the experimental data at ±0.23 Å^−2^ contours (green positive and red negative), and (*c*) fractal dimension plot for the residual potential of the entire unit cell after IAM (blue open circles) and TAAM (red full circles) refinements.

The IAM kinematical refinement for urea on the experimental data was run without any restraints or constraints, analogously as for l-alanine and α-glycine. In the case of urea, the H atoms were also visible on residual electrostatic potential maps (Fig. S7). All ADPs were positively definite (Fig. 8 ▸). The *R*1 and *wR*2 values after IAM were 17.56 and 38.56%, respectively (Table 1 ▸), which were slightly worse than for α-glycine and l-alanine. The residual potential was dominated by negative values [Fig. 9 ▸(*a*)]. Negative peaks were located at covalent bonds, mainly around the N atom. The max/min residual potential values were 0.250/−0.333 Å^−2^, somewhat similar to α-glycine, but closer to zero. The fractal dimension analysis confirmed that the IAM residual potential was biased mostly toward negative values, the left branch of the curve spanned farther away from the zero line (from the −0.12 Å^−2^ value outwards) and departed from the parabolic shape very fast [Fig. 9 ▸(*c*)], and a slight bias on the positive residual potential was also visible.

The *F*_obs_*versus F*_calc_ plot for urea was similar to that for α-glycine, the only difference being that the departure of the most intense reflection from the *F*_obs_ = *F*_calc_ line was not so strong (Fig. S8, IAM).

#### IAM *versus* TAAM fitting

After applying the TAAM refinement against the experimental electron diffraction data for urea, there was an improvement in the fitting quality as compared to IAM (Table 1 ▸). The *R*1 and *wR*2 values became better by 1.28 and 1.47%, respectively. The negative values of the residual potential were much closer to zero [Figs. 9 ▸(*b*) and 9(*c*)], though some small bias toward negative values remained after TAAM refinement. The max/min residual potential values moved toward zero by 0.011/0.018 Å^−2^ after TAAM refinement compared to IAM.

**Figure 10:**
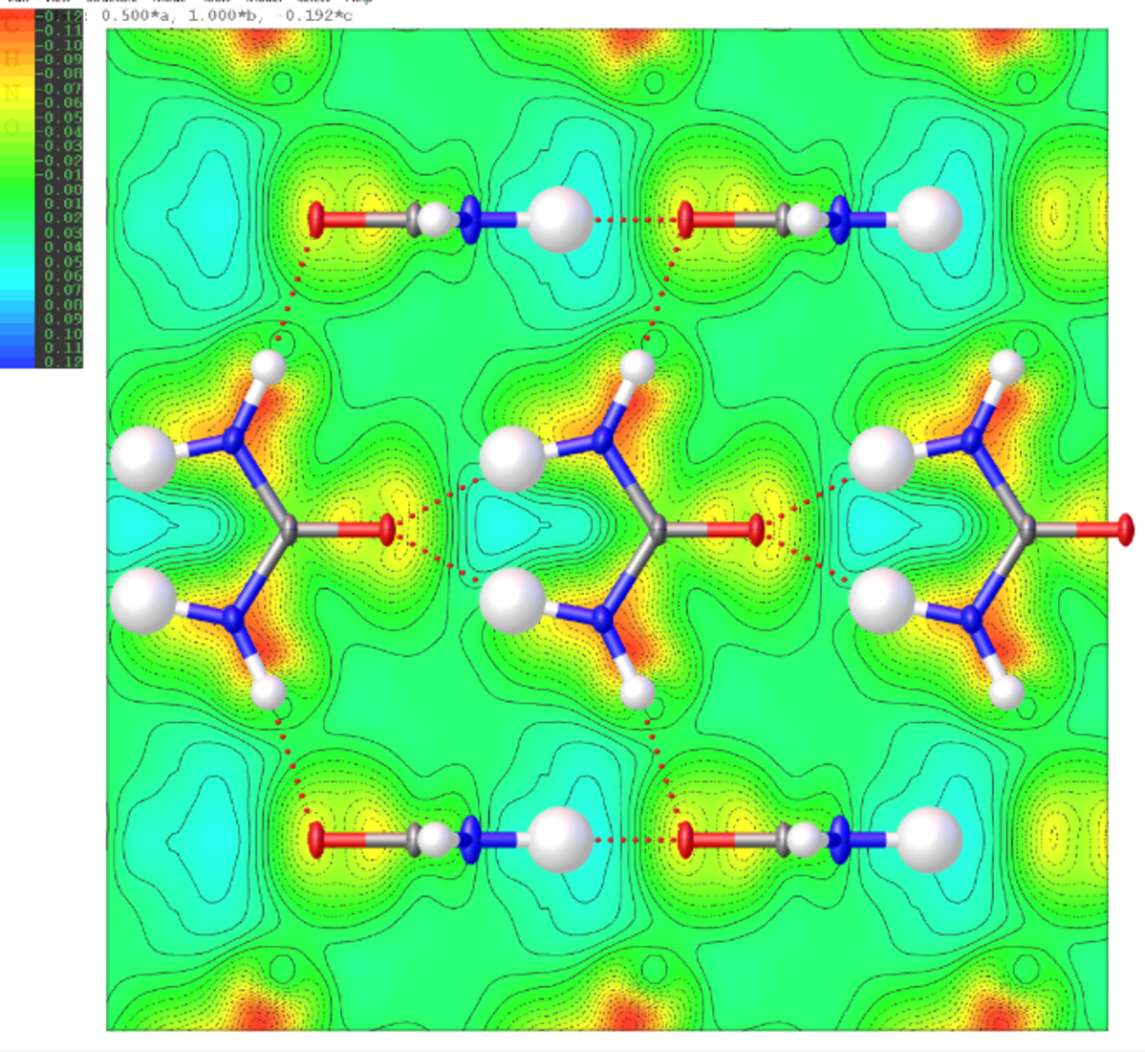
2D Fourier deformation electrostatic potential map (Å^−2^) of the urea crystal computed from TAAM–IAM difference on the structure from experimental TAAM refinement. The 2D map is plotted on the plane passing through all the atoms of the central mol­ecule.

The experimental IAM residual electrostatic potential for urea [Fig. 9 ▸(*a*)] closely resembled the computed deformation electrostatic potential (Fig. 10 ▸). Both potentials exhibited strong negative peaks at the N1—C1 and N1—H1 bond regions, as well as positive peaks between the two symmetry-related H2 atoms. The deformation potential map for urea effectively illustrated the compensation of negative potential resulting from the lone electron pairs at the O1 atom by positive potential generated by the H2 and H1 atoms. Consequently, there were fewer strongly negative residual potential peaks in the vicinity of the O atom in the urea crystal compared to the O atoms in l-alanine and α-glycine; the latter were involved in fewer hydrogen bonds. Another reason could be that the O atom in urea was slightly less negatively charged than the O atoms in alanine and glycine according to the TAAM modelling based on the MATTS pseudoatom data bank.

The *F*_obs_*versus F*_calc_ plots were similar after the IAM and TAAM refinements (Fig. S8), but the TAAM results were usually closer to the *F*_obs_ = *F*_calc_ line, analogously as for l-ala­nine and α-glycine.

Comparing the experimental refinements with the simulated data for urea, it could be concluded that the trends in improving fitting quality while going from IAM to TAAM refinements were very similar. In simulation, the *R*1 value dropped by 1.58% after the TAAM refinement compared to IAM (Table S2). The residual potential was less negative and more featureless when comparing the TAAM to the IAM refinement (Fig. S9). The min/max residual potential values were smaller by 0.013/0.079 Å^−2^ in absolute value. Inter­estingly, after simulated TAAM refinement, the residual potential showed some systematic features not accounted for by TAAM. It was most probably electrostatic potential (electron density) polarization due to neighbouring mol­ecules not modelled by the TAAM parametrized with the MATTS data bank. The experimental data were, obviously, burdened with noise (possibly both random and systematic), as indicated by the significantly wider fractal curves after experimental refinements compared to the very narrow curves observed for the simulated data.

### Structural parameter analysis for l-alanine, α-glycine and urea

TAAM kinematical refinement against 3D ED data led to some changes in the coordinates and ADPs of the structural model compared to IAM.

#### The non-H-atom bond lengths

In the case of the bond lengths between the non-H atoms [Figs. S10(*a*)–(*c*)], the IAM and TAAM refinements on the experimental data for l-alanine, α-glycine and urea led to the values being almost the same, both from a chemical and a statistical point of view. The root-mean-square difference (RMSD) between the experimental results from the IAM and TAAM refinements was only 0.003 Å (Table 2 ▸), which was much smaller than the averaged value of the standard uncertainties (s.u. values) resulting from the least-squares minimiza­tion, the later being equal to 0.017 Å (Table S3). Similar trends were observed for the IAM and TAAM refinements on the simulated data, which showed a very small RMSD of 0.0009 Å, being almost statistically insignificant when compared to the average s.u. values of 0.0005 Å. For both the experimental and the simulated data refinements, the non-H-atom bond lengths were close to the theoretical values resulting from periodic DFT geometry optimization (target values for refinements on the simulated data), with RMSDs of 0.022 and *ca* 0.002 Å for the experimental and simulated data, respectively (Table 2 ▸). Only in the case of the simulated data could the differences be considered statistically significant (*i.e.* were more than three times larger than the s.u. values); however, they were negligible from a chemical point of view (the lengths deviated from the target values differed by only 0.04 to 0.03%).

The experimentally derived non-H-atom bond lengths from this work were similar to the experimental lengths observed in high-resolution X-ray structures (Escudero-Adán *et al.*, 2014 ▸; Aree *et al.*, 2012 ▸; Jha *et al.*, 2020 ▸), and the experimental IAM refinements showed an RMSD of 0.015 Å.

Compared to the published 3D ED data, the l-alanine non-H-atom bond lengths in Khouchen *et al.* (2023 ▸) were 1% longer than in the experimental IAM refinement from this work, but 0.8% shorter than the theoretical DFT values. The non-H-atom bond lengths for the first published α-glycine data (Broadhurst *et al.*, 2020 ▸) were bigger by 2.5 and 1.8% when compared to the experimental IAM refinement and the theoretical DFT values from this work, respectively. Accordingly, the non-H-atom bond lengths from other published work on α-glycine (Klar *et al.*, 2023 ▸) (kinematical refinement) were bigger by 0.2% compared to the experimental IAM re­finement and smaller by 0.7% when compared to the theoretical DFT values from this work. After dynamical re­fine­ment, the values were bigger by 0.1% and smaller by 0.8%. Apparently, dynamical refinement has not influenced greatly the positions of the non-H atoms.

#### The *X*—H bonds

In case of the *X*—H bonds, the experimental TAAM refinement usually led to shorter bonds compared to IAM [Figs. S10(*d*)–(*f*)], with an RMSD of 0.04 Å (Table 2 ▸). The *X*—H bonds from the experimental TAAM refinements were closer by *ca* 0.03 Å to the theoretical values from periodic DFT geometry optimization as compared to experimental IAM refinements; the RMSD values were 0.05 and 0.08 Å for TAAM and IAM, respectively (Table 2 ▸). When compared to experimental average neutron distances (1.033 Å for N^+^—H, 1.099 Å for C*sp*^3^—H, 1.092 Å for C*sp*^3^—H_2_ and 1.077 Å for C*sp*^3^—H_3_) (Allen & Bruno, 2010 ▸) or to the neutron diffraction structure in the case of urea (Swaminathan *et al.*, 1984 ▸), the experimental TAAM refinement values were closer only for l-alanine and urea. For α-glycine, the experimental IAM refinement values were more similar to the average neutron distances. All the above differences observed for the *X*—H bond lengths were, however, on the border of being statistically significant. They were bigger by only 2–4 times than the experimental s.u. values (Table S3), the latter being on average equal to 0.03 and 0.04 Å for the TAAM and IAM refinements, respectively.

The IAM and TAAM refinements on the simulated data showed similar trends for the *X*—H bonds as seen from the experimental data. The simulated IAM *X*—H bonds were usually longer than for TAAM [Figs. S10(*d*)–(*f*)], with an RMSD of 0.012 Å (Table 2 ▸), and were usually longer than their target theoretical values from periodic DFT geometry optimization, with an RMSD of 0.011 Å. The simulated TAAM *X*—H bonds differed from their target values by a similar value (the RMSD was 0.013 Å), but sometimes they were too short and sometimes too long. All the differences were on the border of statistical significance, *i.e.* they were only 2–4 times the s.u. values. The s.u. values were on average equal to 0.004 and 0.003 Å for the simulated IAM and TAAM, respectively.

It is clear, however, that the *X*—H bond lengths from electron diffraction were significantly more accurate than the experimental *X*—H bond lengths from IAM refinements against X-ray diffraction data [Figs. S10(*d*)–(*f*)] (Escudero-Adán *et al.*, 2014 ▸; Aree *et al.*, 2012 ▸; Jha *et al.*, 2020 ▸), the latter method underestimated their values by *ca* 0.18 Å (RMSD between the experimental IAM refinements on the 3D ED and on the X-ray diffraction data).

#### Valence angles for non-H atoms

Valence angles for non-H atoms in all three compounds resulting from experimental IAM and TAAM refinements (Fig. S11) were very similar to each other (differences smaller than the respective s.u. values; Table 2 ▸) and chemically satisfactorily similar to refinements on the simulated data and target values from periodic DFT geometry optimization (differences within 4 s.u.; Table S3). The simulated data confirmed that the IAM and TAAM refinements led to very similar accuracy in valence angle determination.

#### Atomic displacement parameters for non-H atoms

To analyze the atomic displacement parameters, we focused on the *U*_eq_ values for the non-H atoms and the *U*_iso_ values for the H atoms. The *U*_eq_ parameters for the non-H atoms in all three compounds from the experimental IAM refinements were always smaller, by *ca* 4–8%, compared to TAAM [Figs. S12(*a*)–(*c*)], with an RMSD of 0.0009 Å^−2^ (Table 3 ▸). The differences were, however, statistically insignificant; they were within 1 s.u. (Table S4). Similar trends were also visible in the simulated data, with *ca* 3–7% differences and an overall RMSD of 0.00056 Å^−2^. For the simulated data, the differences were on the border of being statistically significant; they were within 3–4 s.u.

By comparing the results of the refinements on the simulated data to their target values, it could be concluded that IAM refinements usually led to smaller values of *U*_eq_ than the expected target values [Figs. S12(*a*)–(*c*)], with an RMSD of 0.00029 Å^−2^, and TAAM refinements led for some atoms to too large and for others to too small values, with an overall RMSD of 0.00041 Å^−2^. All the differences were again statistically on the border of being significant; they were within 3 s.u. The benefits of using TAAM over IAM were almost neglegible for the simulated data from this work; however, they were very visible in the case of the published simulated data for carbamazepine (Gruza *et al.*, 2020 ▸), paracetamol and 1-methyl­uracil (Olech, 2022 ▸). A more extended analysis of the refinements on the simulated data showed that the behaviour of the refined values of the ADPs are strongly dependent on the resolution of the data. Indeed, for a resolution of *d*_min_ = 0.83 Å, *i.e.* worse than in this work, the IAM refinement could lead to *U*_eq_ values for the non-H atoms smaller by 34–74% than their expected target values, whereas the TAAM refinements could lead to values only up to 7% different.

The experimental IAM and TAAM *U*_eq_ parameters for the non-H atoms in l-alanine and α-glycine were, on the other hand, significantly larger, by *ca* 116–124 and 30–40%, respectively, than the experimental reference data from high-resolution X-ray diffraction at 100 K (which were also target values for the simulated data) (Escudero-Adán *et al.*, 2014 ▸; Aree *et al.*, 2012 ▸). For l-alanine, the RMSDs were 0.0132 and 0.0141 Å^−2^ for IAM and TAAM, respectively (Table 3 ▸), and they were more than 16 times larger than the average s.u. values (Table S4). For α-glycine, the RMSD values were much smaller (0.0023 and 0.0031 Å^−2^ for IAM and TAAM, respectively) and on the border of being statistically significant. A somewhat similar situation was seen for the experimental *U*_eq_ values for urea; here the RMSDs had very similar values (0.0023 and 0.0027 Å^−2^) when compared to the reference neutron diffraction data from 123 K (the target values for simulated data) (Swaminathan *et al.*, 1984 ▸) and similarly were on the border of being statistically significant, though for urea the trend that all *U*_eq_ values were larger than for the reference was not observed.

The huge difference for *U*_eq_ for l-alanine is even more strange when we consider the fact that the reference X-ray data were collected at 100 K and the 3D ED experiment in this work was conducted at 81 K; hence, smaller values were expected. Even when compared to the l-alanine structure determined at 150 K by neutron diffraction (Malaspina *et al.*, 2019 ▸), the non-H *U*_eq_ parameters from this work were still larger. Because TAAM refinement against simulated data led to *U*_eq_ parameters differing from their target values by only *ca* ±3%, some effects other than the scattering model must have led to such large 3D ED experimental ADPs, most probably radiation damage and possibly dynamical scattering.

The non-H *U*_eq_ parameters from the published 3D ED data for l-alanine (Khouchen *et al.*, 2023 ▸) were also higher (by 23%) than the reference X-ray diffraction data, but 78% lower than the experimental IAM refinement results presented in this work. The *U*_eq_ values of the non-H atoms for α-glycine from the first published 3D ED data (Broadhurst *et al.*, 2020 ▸) were bigger by *ca* 50 and 170% when compared to the experimental IAM refinement and reference X-ray diffraction values from this work, respectively. Accordingly, the *U*_eq_ values from the second published data (Klar *et al.*, 2023 ▸) (kinematical refinement) were bigger by *ca* 52 and 193% when compared to the experimental IAM refinement and reference values from this work, respectively. After dynamical refinement, the *U*_eq_ values became smaller compared to the kinematical ones and were bigger by *ca* 12 and 55% when compared to the IAM and X-ray reference from this work, respectively. Apparently, the dynamical approach may significantly lower the size of the atomic displacement parameters, but still none of the 3D ED data for l-alanine and α-glycine, from this work or published, reached the values expected for the temperature at which the data were collected.

#### Atomic displacement parameters for H atoms

In the case of the H atoms, the experimental *U*_iso_ parameters from the IAM refinements differed from those of the TAAM refinements by an RMSD of 0.005 Å^−2^, which converts to *ca* 15%, but the difference was either positive or negative [Figs. S12(*d*)–(*f*)] and were statistically insignificant due to being smaller than 1 s.u. (Table S4). The IAM *U*_iso_ parameters for the H atoms from refinements on the simulated data were always smaller than from TAAM, by an RMSD of 0.0035 Å^−2^ (*ca* 13%), the difference being statistically significant (equal to 7 s.u.). The simulated TAAM values were always closer to their target values than IAM; the RMSD values were 0.0023 and 0.0040 Å^−2^, respectively.

Similarly, as for the non-H atoms, the experimental *U*_iso_ values for most of the H atoms in l-alanine [Fig. S12(*d*)] tended to be bigger compared to the reference values estimated by the SHADE method for 100 K, with RMSDs of 0.016 and 0.011 Å^−2^ for IAM and TAAM, respectively, though the relative differences (53 and 39%) were not as large as for the non-H atoms. For α-glycine and urea, the *U*_iso_ values of the H atoms were sometimes bigger and sometimes smaller compared to the reference values estimated by the SHADE method for 100 K in the case of α-glycine and to the reference neutron diffraction data from 123 K (Swaminathan *et al.*, 1984 ▸) in the case of urea [Figs. S12(*e*) and S12(*f*)]. For α-glycine, the *U*_iso_ values differed by an overall RMSD of 0.003 Å^−2^ for both IAM and TAAM, the differences being very small and statisticaly insignificant. For urea, the overall RMSDs were 0.041 and 0.034 Å^−2^ for IAM and TAAM, respectively, which were large but still on the border of being statistically significant if individual s.u. values for the two H atoms in urea were taken into account [Fig. S12(*f*)].

## Conclusions

We collected relatively good quality and complete high-resolution 3D ED data on l-alanine, α-glycine and urea single crystals. We determined the crystal structures of all three compounds *via* the kinematical approach. We performed IAM kinematical refinements against experimental data without any restraints or constraints on the coordinates or the ADPs, including H atoms which were initially visible in Fourier difference maps. After the IAM refinements, all the anisotropic ADPs of the non-H atoms were positively definite for all three compounds.

Residual electrostatic potential maps after the IAM kinematical refinements showed the dominance of negative peaks located mostly at the lone electron pair and bonding regions. Refinements on the simulated data confirmed that the experimental residual potentials qualitatively resembled the expected deformation electrostatic potential.

The TAAM kinematical refinements applied to the 3D ED experimental data visibly improved all fitting statistics compared to IAM, showing that TAAM is a better physical model than IAM. After TAAM refinements, the *R*1 factors decreased and the residual electrostatic potentials were more featureless compared to IAM; in particular, the negative regions were visibly reduced.

The TAAM refinements on the 3D ED data did not improve the accuracy of the bond lengths between the non-H atoms when compared to IAM. The IAM refinements already led to satisfactory accurate non-H-atom bond lengths, though the s.u. values for the experimental data were somewhat large.

The H-atom positions from the IAM kinematical refinements on the 3D ED experimental data were much more accurate when compared to the reference IAM refinements on the X-ray diffraction data. The IAM refinements had, however, the tendency to lead to slightly longer *X*—H bond lengths than TAAM, and TAAM refinements had the potential to further improve the accuracy of these bonds. The experimentally observed differences were, however, of the same magnitude as the s.u. values.

Relatively large s.u. values for structural parameters refined against experimental 3D ED data partially came from very large uncertainities in the determination of the unit-cell parameters. This shows the importance of designing special protocols correcting for geometrical distortions during the 3D ED data reduction specific for particular electron microscopes (Brázda *et al.*, 2022 ▸; Gruene *et al.*, 2022 ▸).

Atomic displacement parameters from the kinematical refinements on the 3D ED experimental data were too large by tens of percent for two of the three studied compounds. Most probably, other, unmodelled, effects were causing this behaviour, such as radiation damage or dynamical scattering.

The study has shown that with the current experimental set-up it is possible to observe details of electrostatic potential deformations due to chemical bonding in organic crystals. The observations qualitatively agree with the simulated data. The deformation signal is strong enough to see the benefits of using a more accurate potential model (TAAM) in achieving a better fit of the model to the experimental data. A more accurate data treatment leading to a more precise determination of the unit-cell parameters and a correction for radiation damage, as well as the application of dynamical dif­fraction theory necessary to achieve more accurate structural parameters, fully benefit from a more accurate TAAM scattering model and performing qu­anti­tative charge density refinement against 3D ED data to extract all the information hidden in the experimental data.

## Data availability

Raw diffraction images and associated data are available online using the following doi: 10.18150/BPNVDX, 10.18150/XCCVDQ and 10.18150/A3WJKN (Dominiak *et al.*, 2024*a* ▸,*b* ▸,*c* ▸) [RepOD (https://repod.icm.edu.pl/), Repository for Open Data, Inter­disciplinary Centre for Mathematical and Computational Modelling, University of Warsaw, Warsaw, Poland]. The CIF files with results from all refinements presented in this work are provided in the supporting information or can be retrieved free-of-charge from the Cambridge Structural Database (CSD) (Groom *et al.*, 2016 ▸) (deposition numbers: CCDC 2361112–2361117).

## Supplementary Material

Crystal structure: contains datablock(s) LAlaIAM, LAlaTAAM, aGlyIAM, aGlyTAAM, UreaIAM, UreaTAAM, global. DOI: 10.1107/S2053229624005357/eq3017sup1.cif

Structure factors: contains datablock(s) . DOI: 10.1107/S2053229624005357/eq3017LAlaIAMsup2.hkl

Structure factors: contains datablock(s) . DOI: 10.1107/S2053229624005357/eq3017LAlaTAAMsup3.hkl

Structure factors: contains datablock(s) . DOI: 10.1107/S2053229624005357/eq3017aGlyIAMsup4.hkl

Structure factors: contains datablock(s) . DOI: 10.1107/S2053229624005357/eq3017aGlyTAAMsup5.hkl

Structure factors: contains datablock(s) . DOI: 10.1107/S2053229624005357/eq3017UreaIAMsup6.hkl

Structure factors: contains datablock(s) . DOI: 10.1107/S2053229624005357/eq3017UreaTAAMsup7.hkl

Supporting information file. DOI: 10.1107/S2053229624005357/eq3017sup8.pdf

CCDC references: 2361117, 2361116, 2361115, 2361114, 2361113, 2361112

## Figures and Tables

**Table 1:** Summary of the data collection, reduction and refinement statistics of the three title compounds

	L-Alanine		α-Glycine		Urea	
Data collection						
Chemical formula	C_3_H_7_NO_2_		C_2_H_5_NO_2_		CH_4_N_2_O	
Tilt angle/tilt speed (°)	0.3/0.6		0.5/1.0		1.0/2.0	
Detector distance (mm)	657					
Temperature (K)	81					
Accelerating voltage (kV)	200					
Wavelength (Å)	0.02508					
						
Data reduction						
Space group	*P*2_1_2_1_2_1_		*P*2_1_/*n*		*P* 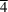 2_1_*m*	
Unit cell *a*, *b*, *c* (Å)	5.89 (7), 5.99 (11), 12.22 (8)		5.11 (10), 11.81 (10), 5.44 (8)		5.596 (3), 5.596 (3), 4.716 (17)	
Angles α, β, γ (°)	90, 90, 90		90, 113.1 (13), 90		90, 90, 90	
Volume (Å^−3^)	431 (10)		302 (9)		147.70 (11)	
Resolution (Å)	0.56		0.56		0.56	
Total reflections	5996		3755		1772	
Unique reflections	2145		1274		457	
Completeness (%)	82.7		71.2		89.9	
Mean *I*/σ(*I*)	7.8		7.1		10.6	
*R*_int_ (%)	16.59		16.61		12.97	
*R*_σ_ (%)	12.88		14.00		9.41	
						
Kinematical refinement	IAM	TAAM	IAM	TAAM	IAM	TAAM
Reflections used [with *I* > 2σ(*I*)]	2145 (1178)		1274 (777)		457 (344)	
Constraints/restraints	0/0		0/0		0/0	
Parameters	83		66		21	
*R*1 [*I* > 2σ(*I*)]	0.1388	0.1313	0.1596	0.1471	0.1756	0.1628
*wR*2 [*I* > 2σ(*I*)]	0.3712	0.3658	0.3578	0.3425	0.3856	0.3709
*R*1 (all data)	0.1907	0.1844	0.2027	0.1917	0.1917	0.1801
*wR*2 (all data)	0.4199	0.4138	0.3931	0.3792	0.4002	0.3866
GooF	1.19	1.13	1.15	1.11	1.39	1.33
Residual potential max/min (Å^−2^)	0.207/−0.216	0.200/−0.212	0.366/−0.434	0.322/−0.329	0.250/−0.333	0.239/−0.315

Computer programs: *CrysAlis PRO* (Rigaku OD, 2024 ▸), *olex2.solve* (Bourhis *et al.*, 2015 ▸), *SHELXT* (Sheldrick, 2015 ▸), *olex2.refine* (Bourhis *et al.*, 2015 ▸) and *OLEX2* (Dolomanov *et al.*, 2009 ▸).

**Table 2:** Root-mean-square difference (RMSD) for non-H atom bond lengths, *X*—H bond lengths and valence angles of L-alanine, α-glycine and urea DFT are the theoretical values resulting from periodic DFT optimization (target values for refinements on simulated data) and XRD are the experimental values resulting from IAM refinements on high-resolution X-ray diffraction data.

RMSD		IAMvsTAAM	IAMvsDFT	TAAMvsDFT	IAMvsXRD
Non-H bond length (Å)	Experimental	0.003	0.022	0.022	0.015
	Simulated	0.0009	0.0020	0.0017	0.0125
*X*—H bond lengths (Å)	Experimental	0.04	0.08	0.05	0.18
	Simulated	0.012	0.011	0.013	0.142
Valence angle (°)	Experimental	0.2	1.3	1.2	0.80
	Simulated	0.10	0.22	0.25	0.60

**Table 3:** Root-mean-square deviation (RMSD) for *U*_eq_ of the non-H atoms and *U*_iso_ of the H atoms in L-alanine, α-glycine and urea ‘Target’ reference values are explained in Table S1 of the supporting information.

RMSD			IAMvsTAAM	IAMvsTarget	TAAMvsTarget
*U*_eq_ (Å^−2^)	Experimental	All compounds	0.0009	0.0088	0.0095
		L-Alanine	0.0010	0.0132	0.0141
		α-Glycine	0.0008	0.0023	0.0031
		Urea	0.0010	0.0023	0.0027
	Simulated	All compounds	0.00056	0.00029	0.00041
*U*_iso_ (Å^−2^)	Experimental	All compounds	0.005	0.019	0.015
		L-Alanine	0.006	0.016	0.011
		α-Glycine	0.002	0.003	0.003
		Urea	0.008	0.041	0.034
	Simulated	All compounds	0.0035	0.0040	0.0023
