# Supplementary material for: TAAM refinement on high-resolution experimental and simulated 3D ED/MicroED data for organic mol­ecules
Source: Acta Crystallogr C Struct Chem. 2024 Jun 27;80(Pt 7):264–77. doi: 10.1107/S2053229624005357 (PMC11225613; doi:10.1107/S2053229624005357)
Supplement: Supplementary file 8 [file c-80-00264-sup8.pdf]

## Supplementary Information

## TAAM refinement on high-resolution experimental and simulated 3D ED/ MicroED data for organic molecules

**Anil Kumar,[1] Kunal Kumar Jha,[1,2] Barbara Olech,[1,2#] Tomasz Goral,[2] Maura Malinska,[3] Krzysztof Woźniak,[2,3] Paulina Maria Dominiak[1]***

1 Biological and Chemical Research Centre, Faculty of Chemistry, University of Warsaw, ul. Żwirki i Wigury 101, 02-089 Warszawa, Poland;

2 Centre of New Technologies, University of Warsaw, ul. S. Banacha 2c, 02-097 Warszawa, Poland;

3 Faculty of Chemistry, University of Warsaw, Pasteura 1, 02-093 Warszawa, Poland

#maiden name: Gruza

**Table S1:** The source of the reference values which were target values for the parameters refined against simulated data.

| **L-alanine** | ***α*-glycine** | **Urea** |
|---|---|---|
| **Atom positions and resulting bond lengths:** | | |
| periodic DFT geometry optimization | periodic DFT geometry optimization | periodic DFT geometry optimization |
| **Non-H atom anisotropic ADPs**: | | |
| IAM refinement on high-resolution X-ray diffraction data collected at 100 K (Escudero-Adán *et al.*, 2014) (CCDC No. 1009312) | IAM refinement on high-resolution X-ray diffraction at 100 K (Aree & Bürgi, 2012) (CCDC No. 849663) | neutron diffraction data collected at 123 K (Swaminathan *et al.*, 1984) (CCDC No. 1278500) |
| **H atom anisotropic ADPs:** | | |
| The SHADE method applied to the structure from the IAM refinement on high-resolution X-ray diffraction collected at 100 K (Escudero-Adán *et al.*, 2014) (CCDC No. 1009312) | The SHADE method applied to the structure from the IAM refinement on high-resolution X-ray diffraction collected at 100 K (Aree & Bürgi, 2012) | neutron diffraction collected at 123 K (Swaminathan *et al.*, 1984) (CCDC No. 1278500) |

**Table S2:** Summary of the simulated data refinements.

| | **L-alanine** | | **α-glycine** | | **Urea** | |
|---|---|---|---|---|---|---|
| *a* (Å) | 5.7890 | | 5.0876 | | 5.5780 | |
| *b* (Å) | 5.9387 | | 11.8092 | | 5.5780 | |
| *c* (Å) | 12.2516 | | 5.4615 | | 4.6860 | |
| *β* (°) | 90 | | 111.992 | | 90 | |
| Volume ($Å^{-3}$) | 421.20 | | 304.254 | | 145.80 | |
| Resolution (Å) | 0.56 | | 0.56 | | 0.56 | |
| Total reflections | 9687 | | 7000 | | 1793 | |
| Unique reflections | 2513 | | 1807 | | 478 | |
| Completeness (%) | 100 | | 100 | | 100 | |
| **Kinematic Refinement** | IAM | TAAM | IAM | TAAM | IAM | TAAM |
| Reflections used (with *I* >2σ(*I*)) | 2513 (2512) | | 1807 (1796) | | 478 (478) | |
| Constraints/ Restraints | 0/0 | | 0/0 | | 0/0 | |
| Parameters | 83 | | 66 | | 21 | |
| *R1* [*I >2σ(I)*] | 0.0380 | 0.0219 | 0.0423 | 0.0277 | 0.0425 | 0.0267 |
| *wR2* [*I >2σ(I)*] | 0.1258 | 0.1037 | 0.2743 | 0.2488 | 0.1375 | 0.1152 |
| *R1* (all data) | 0.0380 | 0.0219 | 0.0424 | 0.0277 | 0.0425 | 0.0267 |
| *wR2* (all data) | 0.1258 | 0.1037 | 0.2755 | 0.2506 | 0.1375 | 0.1152 |
| *GooF* | 0.64 | 0.53 | 1.44 | 1.31 | 0.71 | 0.60 |
| Residual potential Max./min. ($Å^{-2}$) | 0.054/ -0.131 | 0.059/ -0.052 | 0.076/ -0.158 | 0.107/ -0.092 | 0.048/ -0.149 | 0.061/ -0.070 |

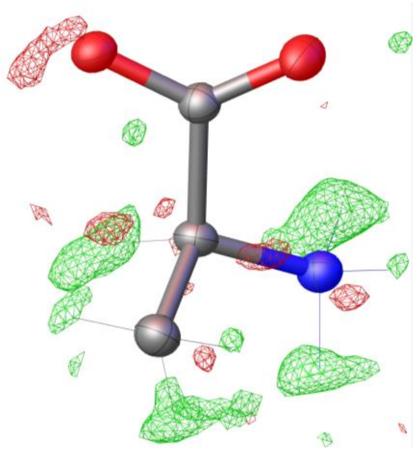

**Figure S1**. Residual potential maps of L-alanine after IAM refinement of non-H atoms against the experimental data at +/- 0.18 Å$^{-2}$ contours (green - positive, red – negative. Residual peacks for all missing hydrogen atoms (indicated by wire frame) are well visible.

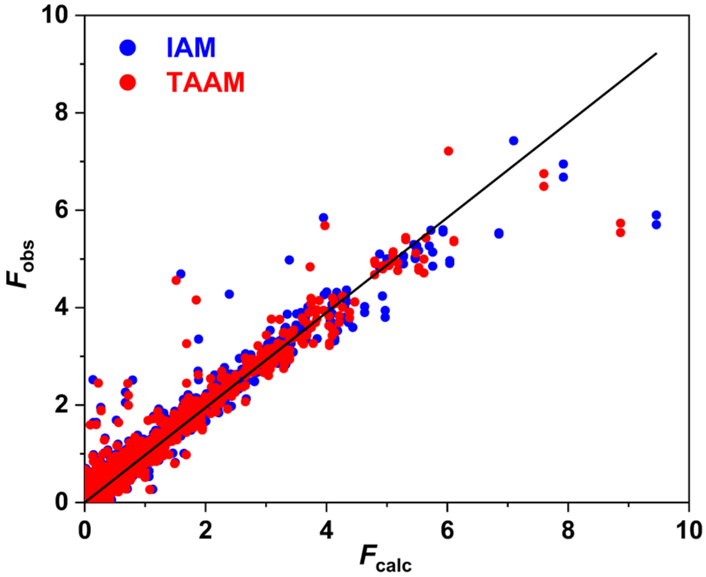


**Figure S2:** Experimental Fobs vs Fcalc plot of L-alanine.

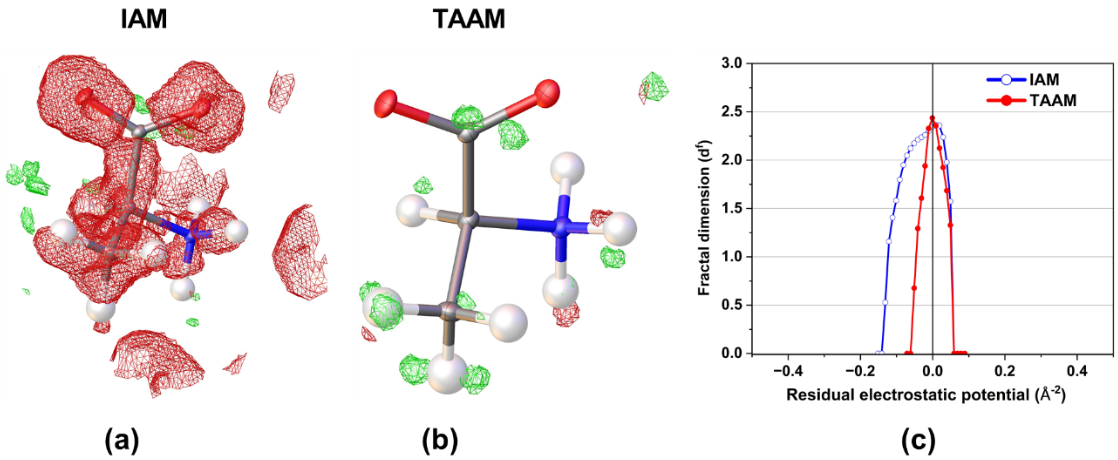


**Figure S3:** Residual potential maps of L-alanine after (a) IAM and (b) TAAM refinement against the simulated electron diffraction data at +/- 0.04 Å$^{-2}$ contours (green positive, red – negative), and (c) fractal dimension plot for residual potential of entire unit cell after IAM (blue open circles) and TAAM (red full circles) refinements.

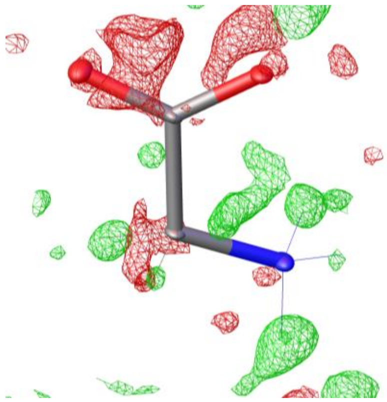

**Figure S4**. Residual potential maps of α-glycine after IAM refinement of non-H atoms against the experimental data at +/- 0.27 Å$^{-2}$ contours (green - positive, red – negative. Residual peacks for all missing hydrogen atoms (indicated by wire frame) are well visible.

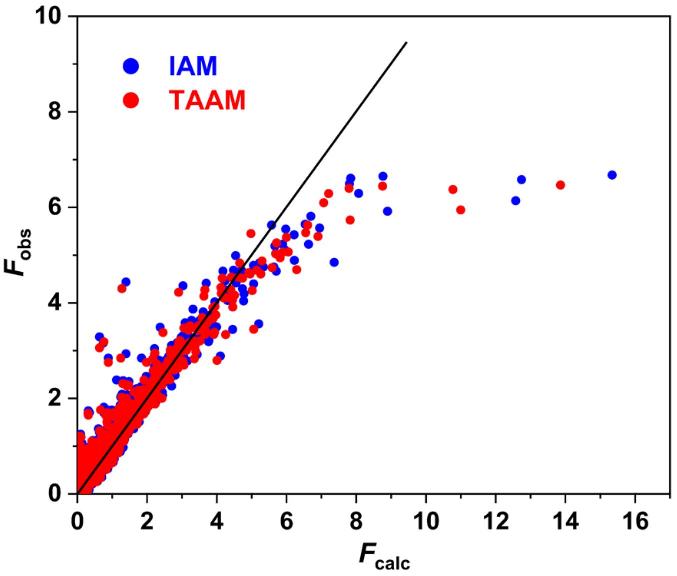


**Figure S5:** Experimental Fobs vs Fcalc plot of α-glycine .

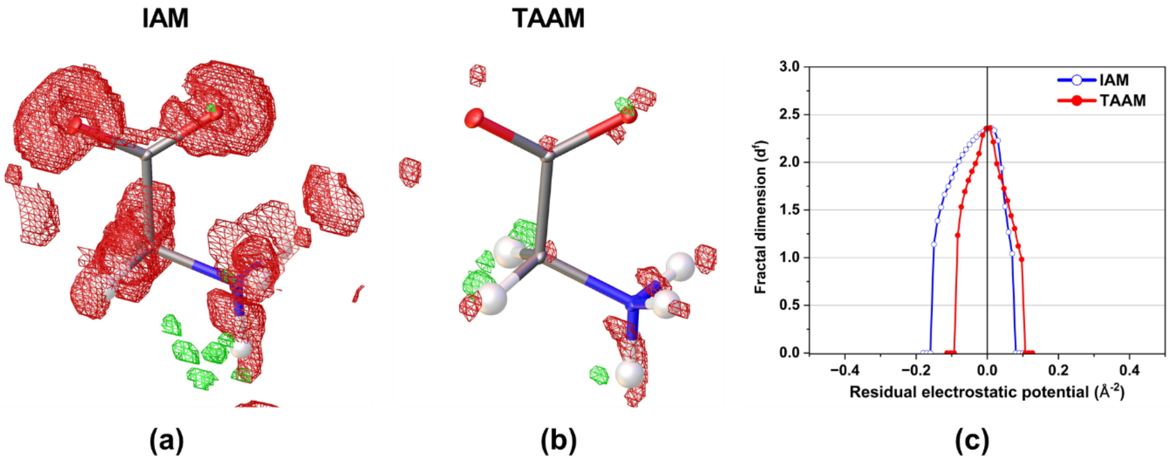


**Figure S6:** Residual potential maps of α-glycine after (a) IAM and (b) TAAM refinement against the simulated electron diffraction data at +/- 0.05 Å$^{-2}$ contours (green positive, red – negative), and (c) fractal dimension plot for residual potential of entire unit cell after IAM (blue open circles) and TAAM (red full circles) refinements.

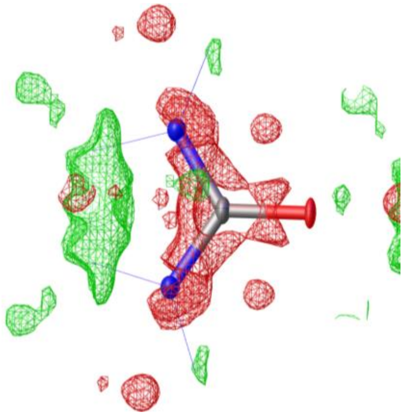

**Figure S7**. Residual potential maps of urea after IAM refinement of non-H atoms against the experimental data at +/- 0.19 Å$^{-2}$ contours (green - positive, red – negative. Residual peacks for all missing hydrogen atoms (indicated by wire frame) are well visible.

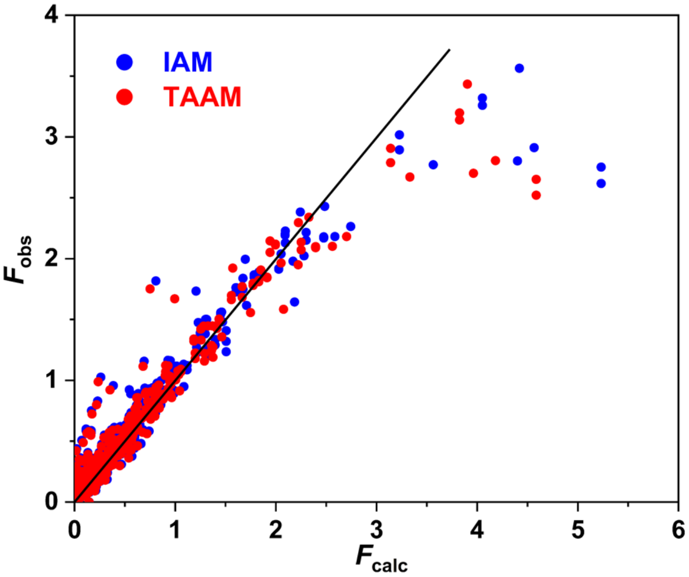


**Figure S8:** Experimental Fobs vs Fcalc plot of urea.

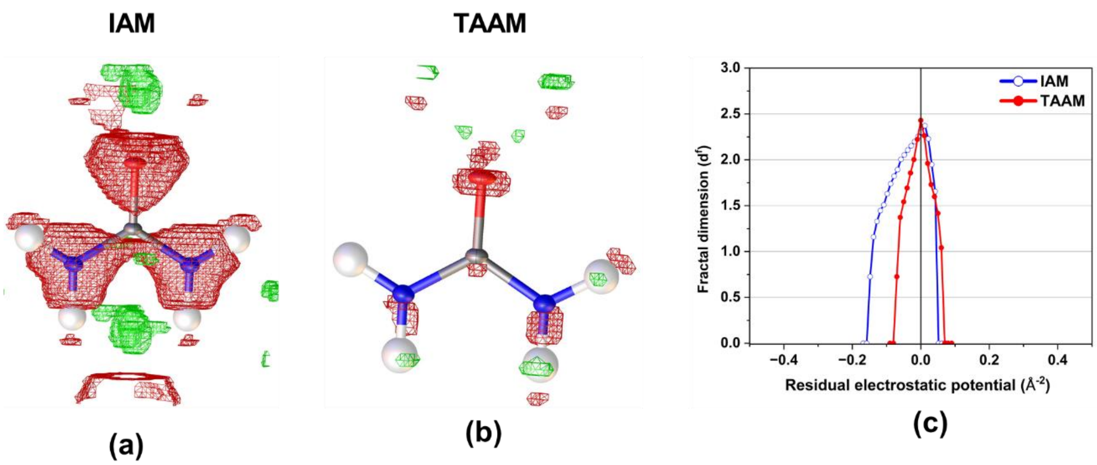


**Figure S9:** Residual potential maps or urea after (a) IAM and (b) TAAM refinement against the simulated data at +/- 0.05 Å$^{-2}$ contours (green - positive, red – negative), and (c) fractal dimension plot for residual potential of entire unit cell after IAM (blue open circles) and TAAM (red full circles) refinements.

**Table S3:** Averaged estimated standard deviation (esd) for non-H atoms bond lengths, X-H bond lengths and valence angles of the l-alanine, glycine and urea.

| esd | | *IAM* | *TAAM* |
|---|---|---|---|
| Non-H bond length (Å) | Experimental | 0.017 | 0.017 |
| | Simulated | 0.0005 | 0.0005 |
| X-H bond lengths (Å) | Experimental | 0.04 | 0.03 |
| | Simulated | 0.004 | 0.003 |
| Valence angle (°) | Experimental | 0.4 | 0.3 |
| | Simulated | 0.03 | 0.03 |

**Table S4 :** Averaged estimated standard deviation (esd) for Ueq and Uiso, X-H of the l-alanine, glycine and urea.

| RMSD | | *IAM* | *TAAM* |
|---|---|---|---|
| Ueq (Å$^{-2}$) | Experimental | 0.0008 | 0.0008 |
| | Simulated | 0.00015 | 0.00013 |
| Uiso (Å$^{-2}$) | Experimental | 0.006 | 0.005 |
| | Simulated | 0.0005 | 0.0005 |

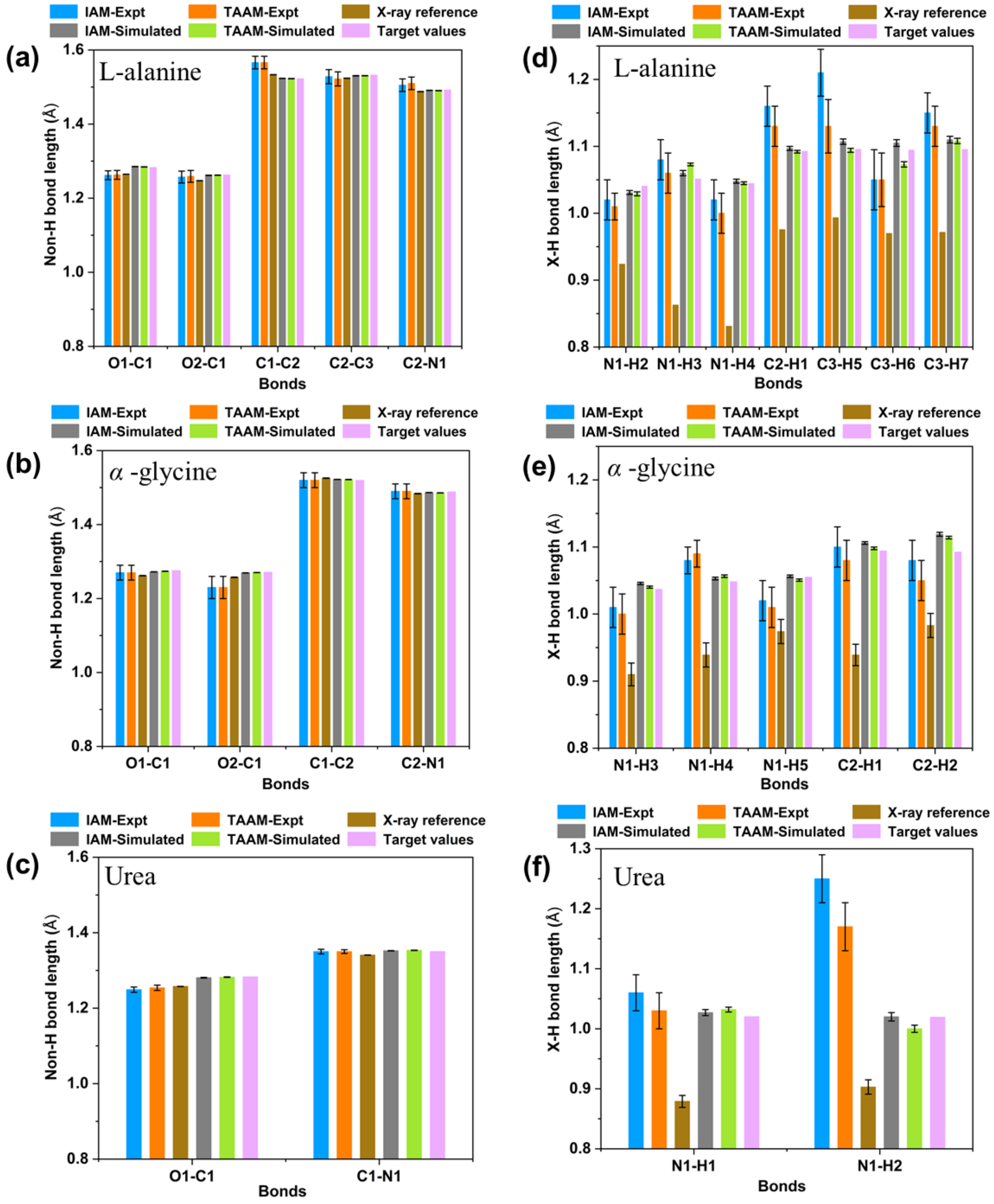


**Figure S10:** Bond lengths with estimated standard deviations for **(a-c)** non-hydrogen atoms (non-H) and **(d-f)** hydrogen atoms (X-H) for **L-alanine**, **α -glycine** and **urea**, respectively from IAM and TAAM refinements against experimental (Expt) and simulated electron diffraction data. X-ray reference – values from X-ray diffraction at 100 K (Escudero-Adán et al., 2014; Aree & Bürgi., 2012; Jha *et al.*, 2020). Target values – values from periodic DFT geometry optimization which were target values for refinements against simulated data.

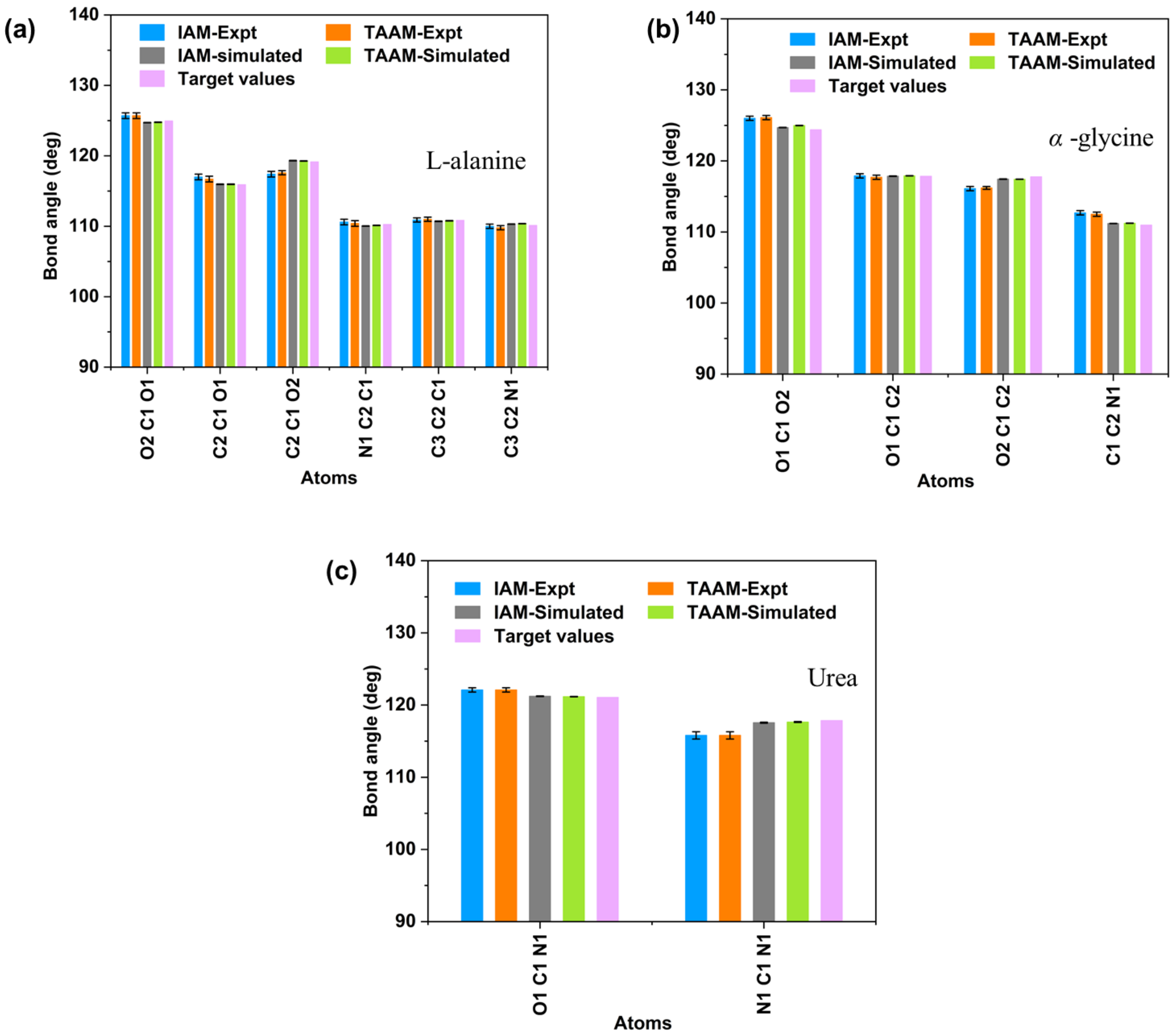


**Figure S11:** Bond angles with standard deviations for (a) L-alanine, (b) α-glycine and (c) urea from IAM and TAAM refinements against experimental (Expt) and simulated electron diffraction data. Target values – values from periodic DFT geometry optimization which were target values for refinements against simulated data

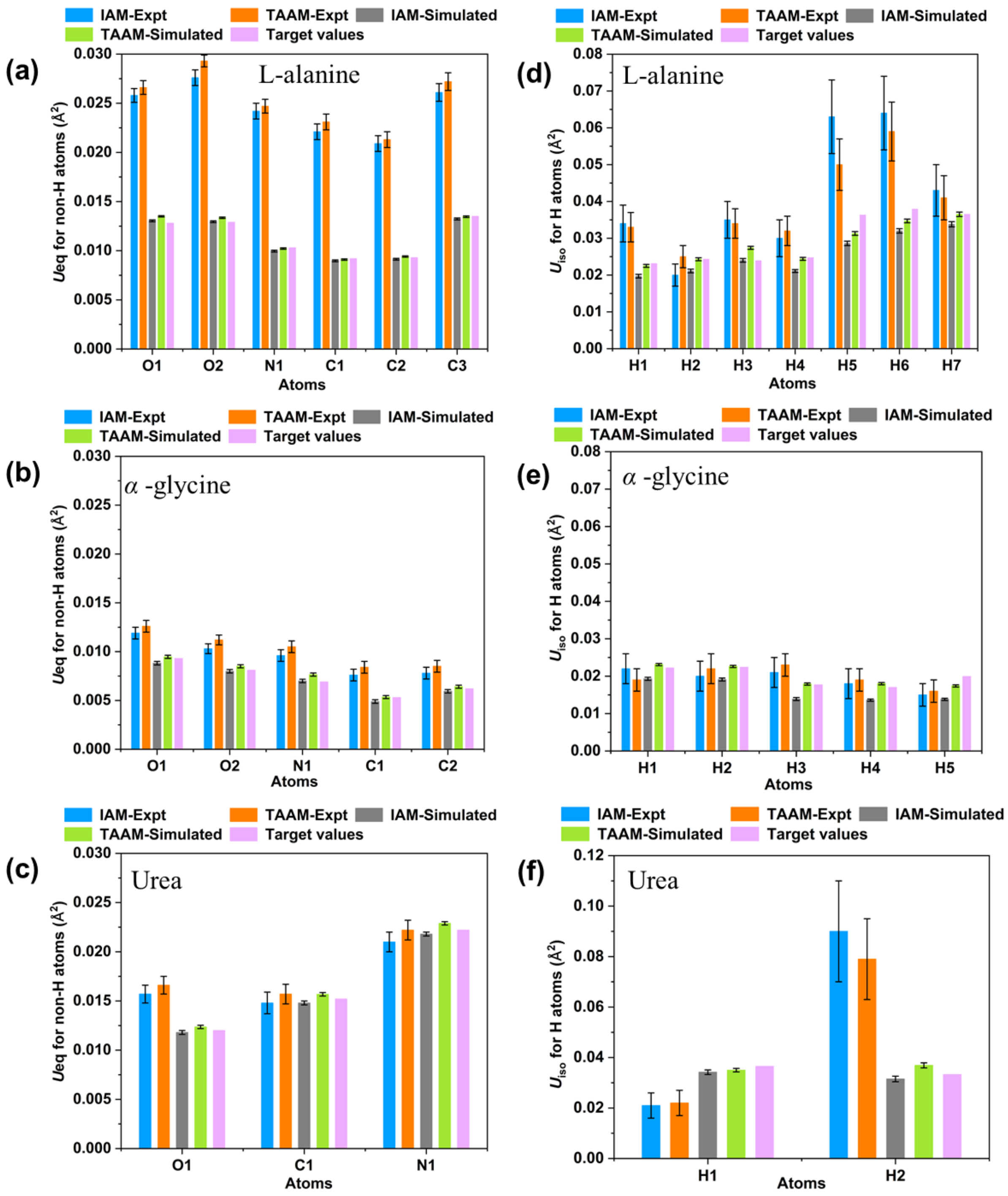


**Figure S12: (a-c)** Ueq with estimated standard deviations for non-hydrogen (non-H) atoms and **(d-f)** Uiso with estimated standard deviations for hydrogen atoms (H) for **L-alanine**, ***α*** **-glycine** and **urea**, respectively from IAM and TAAM refinements against experimental (Expt) and simulated electron diffraction data. Target values for L-alanien and *α* -glycine – for non-H atoms values taken from the reference high-resolution X-ray diffraction from 100 K (Escudero-Adán et al., 2014; Aree & Bürgi., 2012) and for H atoms values taken from the SHADE method, Target values for urea –values taken from neutron diffraction from 123 K (Swaminathan *et al.*, 1984).

## Fractal dimension plot

According to (Meindl & Henn, 2008): “A simple approach to investigate the residual-density distribution is to calculate a histogram and compare it to a Gaussian distribution.” “Owing to the high frequency of residual-density values close to zero, details in the periphery cannot be observed easily”. "The fractal dimension distribution of the residual density is a method which allows to investigate the details of residual density distribution". The method "indicate how much residual density is present and in what way it is distributed, i.e. the extent to which the distribution is featureless". "In the case where only Gaussian noise is present in the residual density, the fractal distribution is parabolic in shape. Deviations from this shape therefore serve as an indicator for systematic errors." "The fractal dimension distribution of the residual density brings all residual-density distributions to one and the same scale" allowing easy comparison of residual densities from various refinements and experiments. For more details, please look (Meindl & Henn, 2008)

## Reference

Meindl, K. & Henn, J. (2008). *Acta Crystallogr. Sect. A Found. Crystallogr.* **64**, 404–418.
